# Effects of Molecular Encapsulation on the Photophysical and Charge Transport Properties of a Naphthalene Diimide Bithiophene Copolymer

**DOI:** 10.1021/acs.chemmater.2c01894

**Published:** 2022-09-05

**Authors:** Stefano Pecorario, Jeroen Royakkers, Alberto D. Scaccabarozzi, Francesca Pallini, Luca Beverina, Hugo Bronstein, Mario Caironi

**Affiliations:** † Center for Nano Science and Technology@PoliMi, Istituto Italiano di Tecnologia, via Giovanni Pascoli 70/3, Milan 20133, Italy; ‡ Department of Energy, Micro and Nanostructured Materials Laboratory—NanoLab, Politecnico di Milano, Via Ponzio 34/3, Milano 20133, Italy; § Sensor Engineering Department, Faculty of Science and Engineering, Maastricht University, P.O. Box 616, 6200 MD Maastricht, The Netherlands; ∥ Department of Chemistry, University of Cambridge, Lensfield Road, Cambridge CB2 1EW, U.K.; ⊥ Cavendish Laboratory, University of Cambridge, Cambridge CB3 0HE, U.K.; # Department of Materials Science, Università di Milano-Bicocca, via Cozzi 55, 20125 Milan, Italy

## Abstract

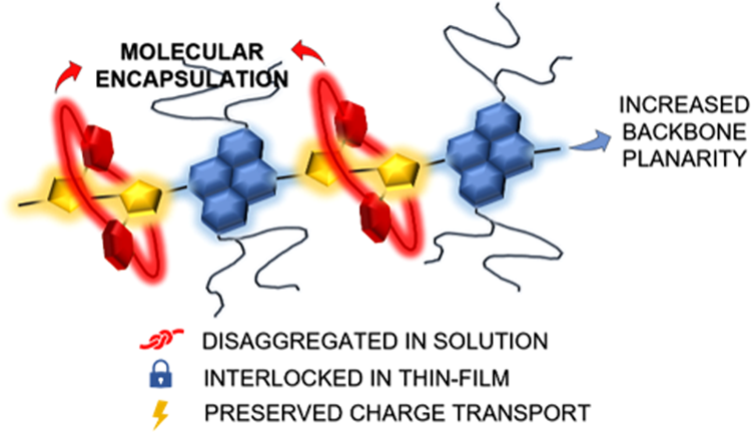

Engineering the molecular structure of conjugated polymers is key to advancing the field of organic electronics. In this work, we synthesized a molecularly encapsulated version of the naphthalene diimide bithiophene copolymer PNDIT2, which is among the most popular high charge mobility organic semiconductors in n-type field-effect transistors and non-fullerene acceptors in organic photovoltaic blends. The encapsulating macrocycles shield the bithiophene units while leaving the naphthalene diimide units available for intermolecular interactions. With respect to PNDIT2, the encapsulated counterpart displays an increased backbone planarity. Molecular encapsulation prevents preaggregation of the polymer chains in common organic solvents, while it permits π-stacking in the solid state and promotes thin film crystallinity through an intermolecular-lock mechanism. Consequently, n-type semiconducting behavior is retained in field-effect transistors, although charge mobility is lower than in PNDIT2 due to the absence of the fibrillar microstructure that originates from preaggregation in solution. Hence, molecularly encapsulating conjugated polymers represent a promising chemical strategy to tune the molecular interaction in solution and the backbone conformation and to consequently control the nanomorphology of casted films without altering the electronic structure of the core polymer.

## 1 Introduction

Conjugated polymers are an important class of materials that have widespread applications, ranging from organic light-emitting diodes (OLEDs),^1^ organic photovoltaics (OPVs),^2−6^ and organic field-effect transistors (OFETs),^7,8^ to energy storage,^9,10^ neuromorphic devices,^11,12^ and sensing.^13−15^ Their great optoelectronic performance originates from their π-conjugated structure, which enables charge/exciton transport and the absorption or emission of energy. Narrow-band-gap materials are of particular interest given that they can capture a great proportion of incident light (e.g., in OPVs) or emit in biologically relevant optical windows (e.g., for imaging).^16^ Owing to their π-conjugated backbone, polymeric chains have a high propensity to aggregate via π–π stacking. Aggregation can be beneficial for certain thin film devices (by promoting intermolecular charge carrier hopping or charge delocalization),^8,17^ while in some cases, it may be unfavorable for the photophysical properties (e.g., photoluminescence (PL)) in condensed phases.^16,18−24^ The development of semiconducting polymers that preserve their conjugated backbone from aggregation, while offering more control over intermolecular interactions and processes, remains extremely challenging.

Molecular encapsulation is a powerful, synthetic concept that can be used to study polymer chains (or molecules) in isolation by shielding the conjugated backbone with protective macrocycles and preventing electronic cross-communication between the π-systems.^16,18−21,25−30^ Noncovalently threaded polyarylene-based conjugated polymers,^31^ along with covalently encapsulated thiophene/phenyl-based^32^ and diketopyrrolopyrrole (DPP)-based conjugated polymers,^16^ have demonstrated superior photoluminescence and OLED external quantum efficiencies compared to the reference polymers. Thus, even though molecularly encapsulated conjugated polymers display reduced charge transport properties by the suppression of intermolecular hopping, they can afford high-performance optoelectronic devices, an area that has been underexplored.

Naphthalene diimide (NDI)-based π-conjugated polymers have become one of the most heavily studied conjugated polymers for electron transport as they exhibit good electronic properties in relevant optoelectronic devices, such as high charge carrier mobility in n-type OFETs and as non-fullerene acceptors in OPV blends.^33−35^ By preparing a molecularly encapsulated version of the most well-known polymer (i.e., PNDIT2 or otherwise known as P(NDI2OD-T2)) in this study, we can therefore probe the effect of molecular encapsulation on its photophysical and electron transporting properties. We recently reported a molecular encapsulated NDI–thiophene-based conjugated polymer where the naphthalene diimide portion of the molecule was encapsulated, leading to lower photoluminescence quantum yield with respect to the reference polymer.^19^ Here, we present the molecular encapsulation of the bithiophene portion of PNDIT2, aiming to control the extent of intermolecular interactions by leaving the naphthalene diimide portion of the conjugated polymer available for intermolecular interactions. A thorough spectroscopic analysis of the absorption and photoluminescence reveals that the molecular encapsulation of PNDIT2 promotes a planarization of the polymer backbone and strongly controls the packing of the polymer both in solution and in thin film. Strikingly, the encapsulating rings covalently bonded to the thiophene units prevent the preaggregation of the polymer chains in common organic solvents. Still, they permit π-stacking between the NDI moieties in solid state, preserving charge transport within the polymer network (field-effect mobility of ∼10^–3^ cm^2^ V^-1^ s^-1^). Thus, we believe that these new molecularly encapsulated materials may hold the key to advancing optoelectronic devices, allowing control over their intermolecular interactions without compromising other optoelectronic properties.

## 2 Results and Discussion

### 2.1 Synthesis

**Scheme 1:**
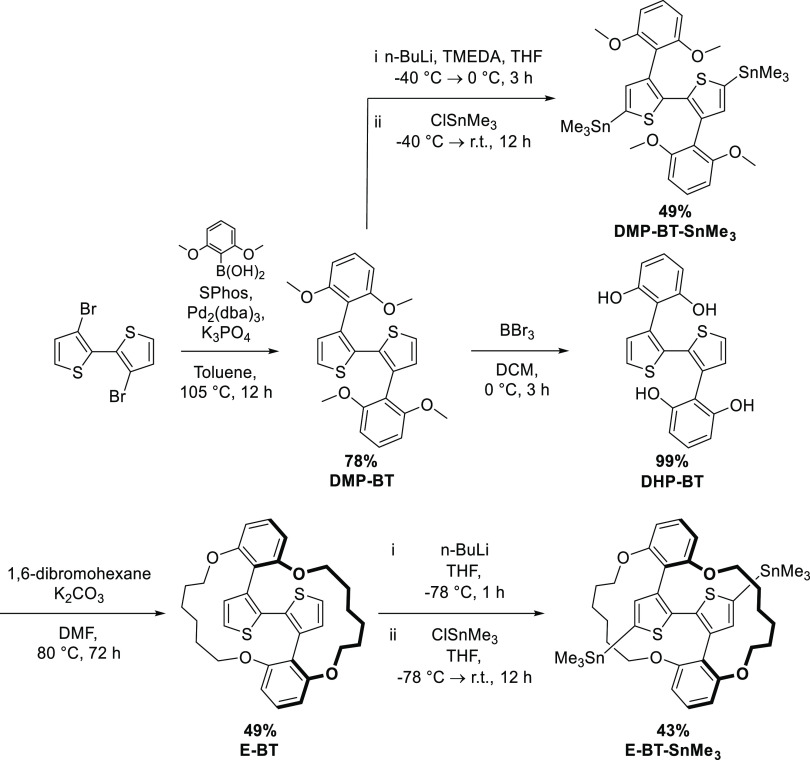
Overall Synthesis of Monomers DMP-BT-SnMe_3_ and E-BT-SnMe_3_

**Scheme 2:**
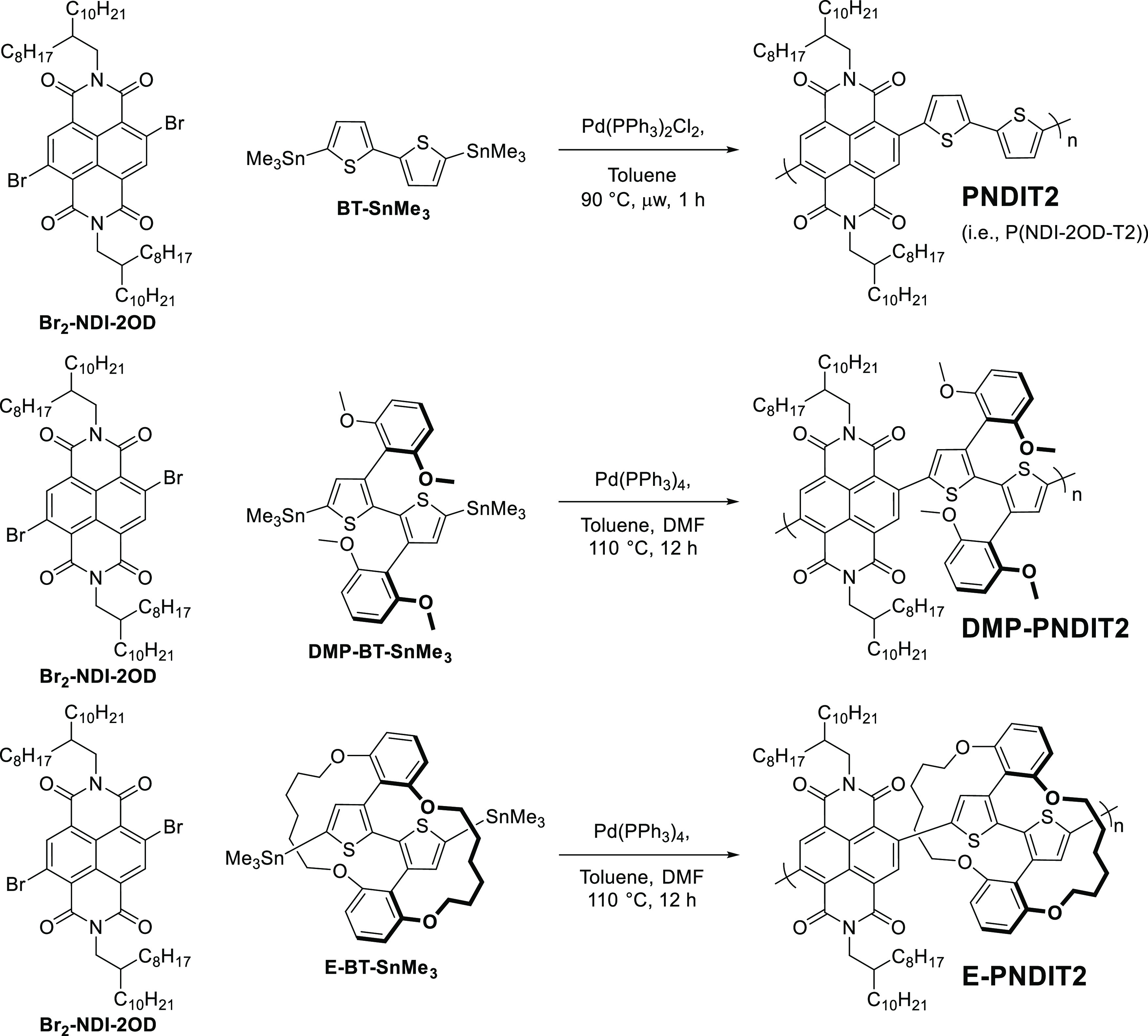
Overall Synthesis of Polymers PNDIT2, DMP-PNDIT2, and E-PNDIT2

Molecularly encapsulated bithiophenes have been reported previously by Sugiyasu and coauthors.^30^ In our work, we simplified the synthesis of encapsulated bithiophenes by eliminating the need for separate alkylation and metathesis steps. The synthesis of the bithiophene monomers is illustrated in Scheme 1. 3,3-Dibromo-2,2-bithiophene was reacted with 2,6-dimethoxyphenylboronic acid in a Suzuki–Miyaura cross-coupling reaction to afford DMP-BT in a 78% yield. Upon further stannylation (via lithiation), the DMP-BT-SnMe_3_ monomer was generated in a 49% yield after purification. Next, DMP-BT was demethylated with BBr_3_, encapsulated with 1,6-dibromohexane, and stannylated to form the final E-BT-SnMe_3_ monomer. Lastly, the commercial 5,5′-bis(trimethylstannyl)-2,2′-bithiophene (BT-SnMe_3_), bulky DMP-BT-SnMe_3_, and encapsulated E-BT-SnMe_3_ were each separately copolymerized with Br_2_-NDI-2OD (based on previous literature procedures^7,36,37^) to generate PNDIT2, DMP-PNDIT2, and E-PNDIT2, respectively (Scheme 2). By comparing their photophysical, microstructural, and charge transport properties, we aim to elucidate the effect of the encapsulating rings in E-PNDIT2, with respect to both the reference PNDIT2 and the bulky DMP-PNDIT2 analogs.

**Figure 1:**
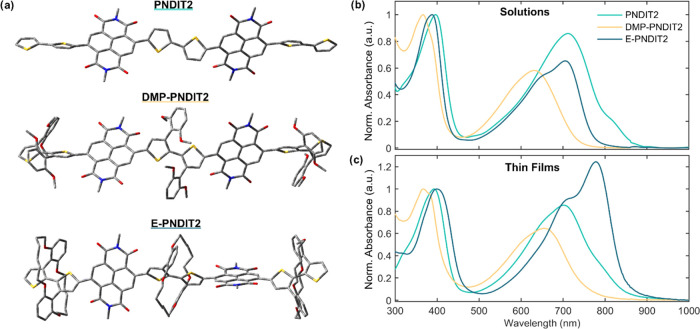
(a) DFT-optimized structures (B3LYP/6-31G*; details in the SI) of PNDIT2, DMP-PNDIT2, and E-PNDIT2 fragments. Side chains are not displayed. (b) UV–vis absorption spectra of PNDIT2, DMP-PNDIT2, and E-PNDIT2 dissolved in toluene at a concentration of 0.1 g/L and (c) in thin film. The spectra are normalized at the π–π* transition peaks. The unnormalized and normalized absorption spectra plotted as a function of the photon energy are reported in Figure S3.

All polymers were obtained with molecular weights *M*_n_ > 19 kDa and dissolved in common organic solvents. The physical properties are summarized in Table 1. The density functional theory (DFT)-optimized structures for the series of polymers are shown in Figure 1a. The calculated dihedral angles, highest occupied molecular orbital (HOMO)/lowest unoccupied molecular orbital (LUMO) energetic levels, and spatial distributions are provided in the Supporting Information (SI).

**Table 1:** Physical Properties of the NDI-Based Conjugated Polymers

polymer	*M*_n_ (kDa)^a^	*M*_w_ (kDa)^b^	dispersity^c^	*X̅*_n_^d^	*X̅*_w_^e^	*E*_g_ (eV)^f^
PNDIT2 (commercial)	35.3	63.5	1.80	36.8	66.3	1.98
PNDIT2 (synthesized)	45.0	136.1	3.02	47.0	142.1	1.98
DMP-PNDIT2	19.4	37.1	1.91	15.7	30.1	1.85
E-PNDIT2	19.9	46.3	2.33	14.9	34.6	1.80

a Number-average molecular weight.

b Weight-average molecular weight.

c *M*_w_/*M*_n_.

d Number-average degree of polymerization (average number of monomer units per polymer chain).

e Weight-average degree of polymerization.

f HOMO–LUMO energy gap, calculated by DFT using B3LYP/6-31G*.

The thermal properties of the newly synthesized DMP-PNDIT2 and E-PNDIT2 polymers were investigated by thermogravimetric analysis (TGA) and differential scanning calorimetry (DSC), and the results are reported in Figures S1 and S2, respectively. Both polymers are thermally stable up to 400 °C. Interestingly, the DSC measurement of E-PNDIT2 shows a broad exothermic peak between 140 and 250 °C in the first heating cycle. This feature, which is not observed for PNDIT2 and DMP-PNDIT2, is related to a crystallization phenomenon driven by interpenetration of the encapsulating rings, as revealed based on diffraction measurements in the next sections.

### 2.2 Optical Properties

The UV–vis absorption spectra of PNDIT2, DMP-PNDIT2, and E-PNDIT2 in solution (0.1 g/L in toluene) and in thin film are shown in Figure 1b,c, respectively. As for other donor–acceptor copolymers,^38−42^ the absorption spectra are characterized by a high energy band ascribed to the π–π* transition and by a low-energy band corresponding to the intramolecular charge-transfer (CT) transition, which reflects a redistribution of the charge density along the polymer chain from the donor moiety (i.e., the T2 unit) to the acceptor one (i.e., the NDI unit). The spectral features of PNDIT2 have been established previously.^38,43,44^ It is well known that PNDIT2 forms aggregates when dissolved in toluene and other common organic solvents, except for chloronaphthalene.^38,44,45^ Such a preaggregation phenomenon is not due to the interchain stacking but arises from coiling within the single polymer chain, as proven by ultracentrifugation measurements and by the robustness of the CT spectral features when comparing concentrated and diluted solutions.^38^ Hence, the CT band in solution results from the convolution of semicrystalline and amorphous phases and its fine structure varies depending on the solvent and temperature. More specifically, the peak at 710 nm and the shoulder at ∼800 nm correspond to two distinct aggregation states, while the broad band centered at ∼620 nm originates from unaggregated chains.^38,43^ The solid-state spectrum of PNDIT2 resembles the one in solution, with only a slight remodulation of the contributions of the semicrystalline and amorphous phases to the CT band.

The absorption spectra of DMP-PNDIT2, in both solution and thin film, are blue-shifted with respect to PNDIT2 and present a broad CT band centered at 630 nm. Such features resemble the spectrum of unaggregated PNDIT2 in chloronaphthalene solution, in which preaggregation upon coiling of the polymer chain does not occur.^38^ Therefore, the absorption features of DMP-PNDIT2 suggest that the dimethoxyphenyl groups likely prevent the preaggregation of the polymer chains in solution. Consequently, unaggregated polymer chains abound in solution and the amorphous phase prevails in solid state. In E-PNDIT2, the encapsulating rings enforce coplanarity within the bithiophene units, hence extending the effective conjugation length of the polymer backbone.^30,46,47^ The solution absorption spectrum of E-PNDIT2 is therefore red-shifted with respect to that of DMP-PNDIT2. The π–π* transition peak is located at 388 nm with a shoulder at ∼374 nm, while the low-energy band, corresponding to the CT transition, exhibits an evident fine structure with a main peak at 706 nm and a secondary one at ∼650 nm. Similar to PNDIT2, such a structured CT band might suggest the presence of preaggregation in solution as the two peaks represent either an amorphous or aggregated phase. However, this study provides evidence that this is not the case. First, the relative intensity and wavelength of the absorption peaks are not concentration-dependent (Figure S4), meaning that interchain aggregation is not occurring. Second, the absorption spectrum is insensitive to the solution temperature, as shown in Figure S5. This provides clear evidence that neither interchain nor intrachain aggregation (coiling) occurs in solution. In fact, the amount of aggregated states would diminish with increasing temperature and result in a change of the absorption spectrum, as observed for PNDIT2.^38^

Figure S6 displays how the absorption of E-PNDIT2 varies when dissolved in a different solvent. The main CT peak and the absorption tail show a clear bathochromic shift with increasing solvent polarity. Except for solvatochromism, there is no neat variation of the absorption features upon solvent change. This contrasts with the behavior of PNDIT2, whose aggregation is strongly affected by the solvent choice. It is worth mentioning that the polymer molecular weight plays an important role in determining the amount of intrachain aggregation at a given concentration, with longer chains (>80 repeating monomer units) being more prone to coiling.^44,45^ Based on UV–vis spectroscopy, Nahid and coauthors^48^ provided evidence that PNDIT2 with the number-average degree of polymerization *X̅*_n_ = 7 is completely unaggregated in dichlorobenzene solution. Instead, starting from *X̅*_n_ = 13.6, they observed a systematic increase in the coiling of the polymer chains with the molecular weight. In this work, DMP-PNDIT2 and E-PNDIT2 have respective values of *X̅*_n_ = 15.7 and 14.9 (Table 1), which is above the limit for aggregation in the case of PNDIT2.

**Figure 2:**
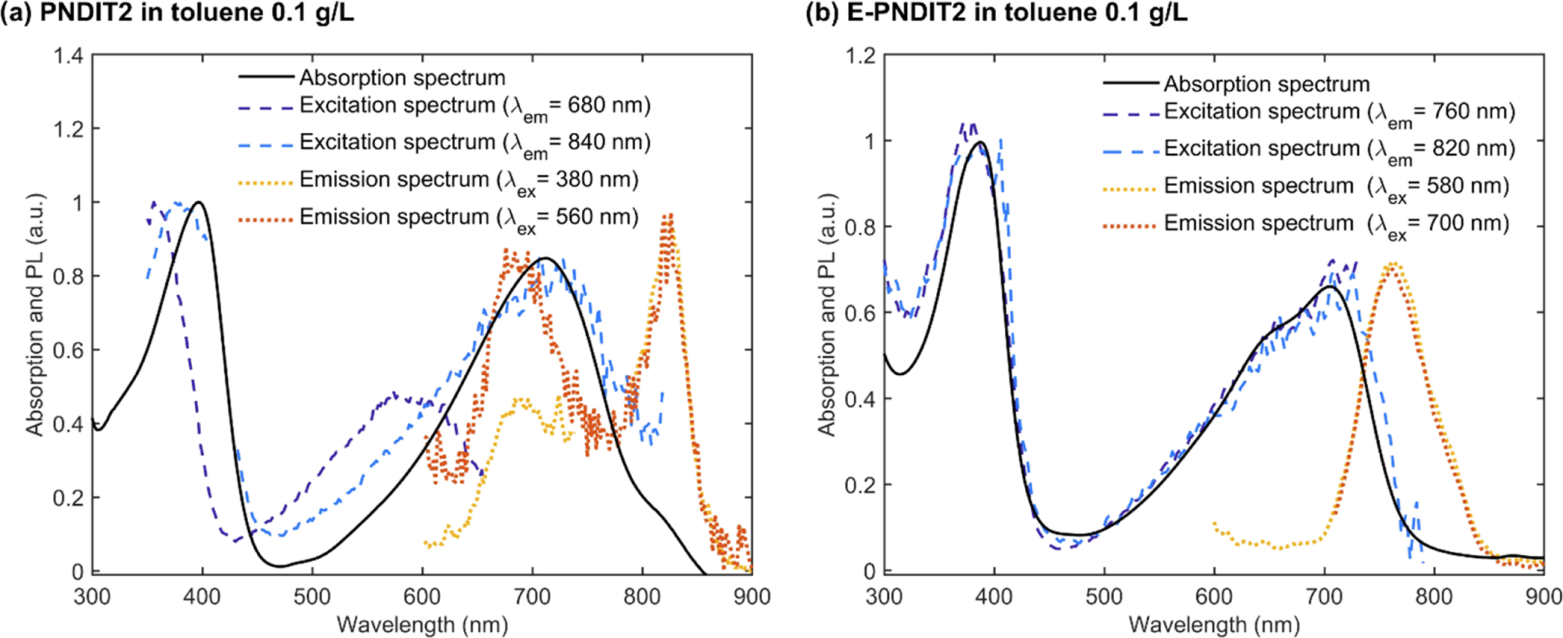
Photoluminescence emission and excitation spectra of (a) PNDIT2 and (b) E-PNDIT2. Both the polymers are dissolved in toluene at a concentration of 0.1 g/L.

Further evidence of the different behavior of PNDIT2 and E-PNDIT2 in solution is provided by photoluminescence spectroscopy. The emission and excitation spectra of PNDIT2 and E-PNDIT2 are reported in Figure 2a,b, respectively. In agreement with the findings by Steyrleuthner and coauthors,^38^ the emission spectrum of PNDIT2 is characterized by two peaks at ∼680 and ∼825 nm, which are assigned to the emission of the unaggregated and aggregated phases, respectively. Indeed, the relative intensity of these two peaks depends on the excitation wavelength (orange and red curves). In addition, the excitation spectra corresponding to the emission at 680 and 840 nm (dark blue and light blue curves, respectively) do not resemble the absorption spectrum (black curve). In contrast, E-PNDIT2 displays only one emission peak at 760 nm with a shoulder at ∼820 nm, irrespective of the excitation wavelength (Figures 2b and S7). Moreover, the acquired excitation spectra in correspondence with the emission at 760 and 820 nm (dark blue and light blue curves, respectively) are identical and resemble the absorption spectrum (black curve), proving that the fine structure in the absorption and photoluminescence spectra of E-PNDIT2 should not be ascribed to different aggregates but to a vibronic progression of unaggregated polymer chains (further details are provided in Figure S8).^49−52^ This has also been shown for other molecularly encapsulated polymers as molecular encapsulation can increase backbone rigidity and suppress rotational and vibrational degrees of freedom.^16^ Both the photoluminescence spectra of E-PNDIT2 and PNDIT2 thin films (Figure S9) present a unique peak at ∼820 nm, suggesting that the aggregated phases of PNDIT2 and E-PNDIT2 share a similar electronic structure.

Finally, the absorption spectrum of E-PNDIT2 in thin film displays a rigid red shift with respect to the absorption in solution, which is due to the formation of ordered semicrystalline phases in the solid state. Such a rigid shift would not be possible if the polymer chains were already preaggregated in solution (like for PNDIT2) or if the chains form amorphous domains, as in the case of DMP-PNDIT2. All of these arguments lead to the conclusion that E-PNDIT2 chains are not aggregated in solution. Hence, the bathochromic shift with respect to DMP-PNDIT2 indicates a planarization of the polymer backbone, resulting in a more extended delocalization of the electron density within the polymer chain. In the solid state, ordered semicrystalline phases are forming, as discussed further below. The encapsulating rings of different polymer chains interact with each other, controlling both the intermolecular packing and the intramolecular electronic structure. Indeed, the planarization effect induced by the encapsulating rings becomes even stronger upon aggregation in thin film, likely due to an intermolecular-lock mechanism.^21,53^ Thus, a comprehensive rigidification of the backbone occurs as a consequence of the planarization of both the T–T and T–NDI torsional angles. This is testified by the rigid red shift of the absorption spectrum from solution to thin film and by an increase in the intensity of the CT band with reference to the π–π* peak.

### 2.3 Thin Film Microstructure

Several studies highlighted the impact of PNDIT2 preaggregation in solution on the microstructural properties of thin films formed upon solution casting.^43,54−57^ On the one hand, preaggregation is a key requirement to achieve thin films with improved energetic order and with a preferential alignment of the polymer chains, resulting in superior charge transport properties.^55,56,58,59^ On the other hand, the formation of crystalline clusters can be detrimental for applications requiring an interpenetrating network of different materials, as in the case of donor and acceptor polymers in bulk heterojunction solar cells^44,60,61^ or of doped blends based on organic semiconductors for thermoelectric applications.^62,63^

**Figure 3:**
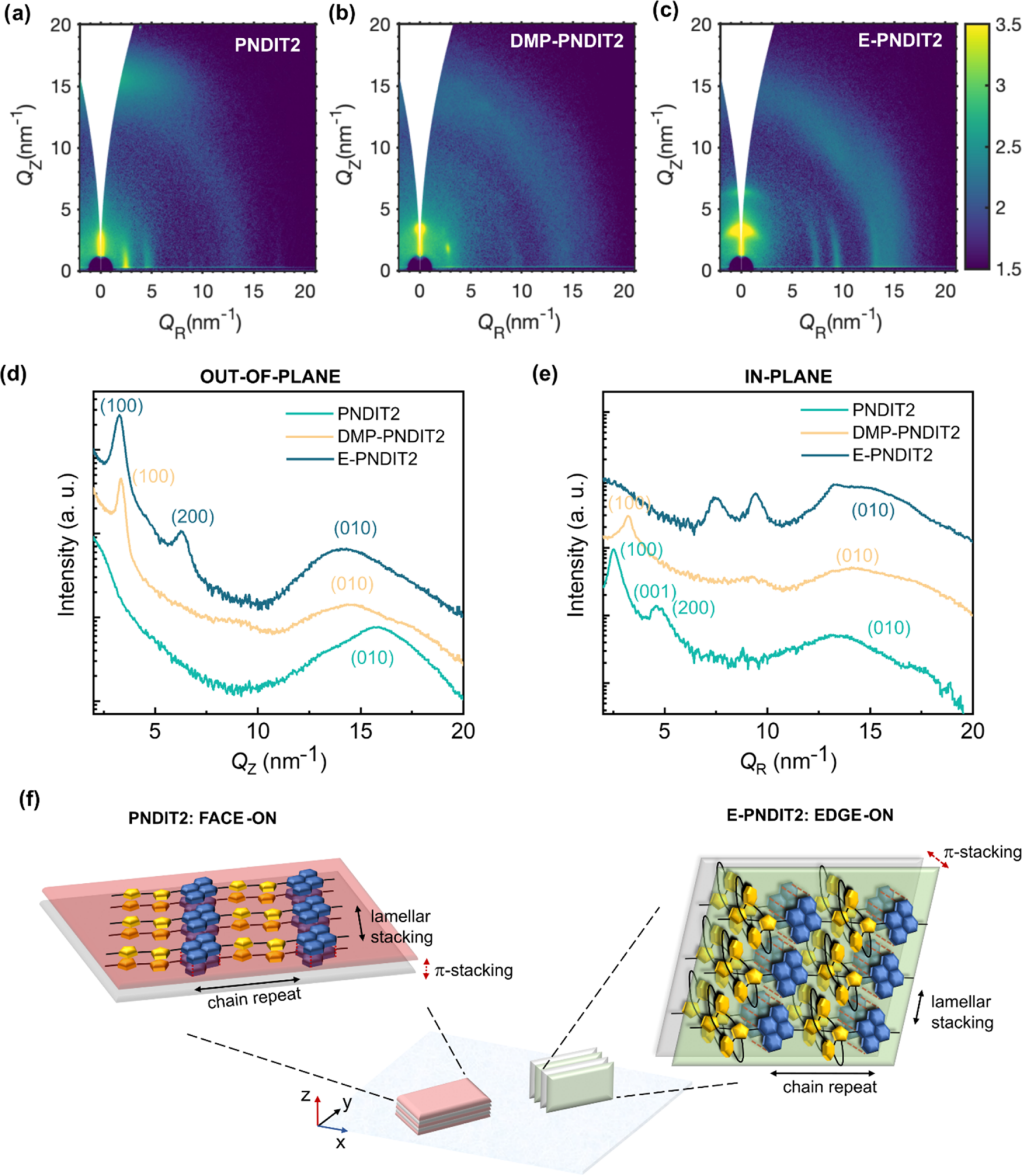
GIWAXS diffraction patterns of thin films of (a) PNDIT2, (b) DMP-PNDIT2, and (c) E-PNDIT2, which were deposited by off-center spin-coating from a toluene solution at a concentration of 5 g/L and annealed for 30 min at 180 °C. Diffractograms of the three polymers integrated along (d) the out-of-plane direction and (e) the in-plane direction. (f) Cartoon depicting the face-on texture characteristic of PNDIT2 films and the edge-on molecular stacking of E-PNDIT2 films.

To investigate the effect of molecular encapsulation on the microstructure, we performed grazing-incidence wide-angle X-ray scattering (GIWAXS) on films of PNDIT2, DMP-PNDIT2, and E-PNDIT2. All of the films were spin-casted from a toluene solution at a concentration of 5 g/L and then annealed at 180 °C for 30 min. The bidimensional diffraction patterns of the three polymers are displayed in Figure 3a–c. The one-dimensional profiles along the out-of-plane (OOP) and in-plane (IP) scattering directions are reported in Figure 3d,e, respectively. The diffraction pattern of PNDIT2 (Figure 3a) is in very good agreement with previous reports.^48,64^ The film presents a preferential face-on packing, as schematically depicted in the cartoon in Figure 3f. Indeed, the (100) and (001) peaks, corresponding to the lamellar stacking and to the chain backbone repeat, are oriented in the in-plane direction, while the broad (010) peak associated with the π-stacking of the NDI moieties is oriented in the out-of-plane direction. The diffraction pattern of DMP-PNDIT2 indicates a semicrystalline microstructure (Figure 3b). However, compared to PNDIT2, the scattering features are less sharp, meaning that the degree of crystallinity of the film is lower. Strikingly, the pattern of E-PNDIT2 presents sharp and oriented diffractions, implying higher semicrystallinity with respect to DMP-PNDIT2 and revealing a preferential edge-on orientation, as illustrated in the cartoon in Figure 3f.

A complete assignment of the diffraction features of DMP-PNDIT2 and E-PNDIT2 would require resolving their single-crystal structure. However, a close comparison with PNDIT2 offers the possibility to assign most of the diffraction peaks (Figure S10). The *Q* vectors and interplanar spacings corresponding to the (100), (001), and (010) diffraction peaks of the thin films of the three polymers are listed in Table 2. DMP-PNDIT2 and E-PNDIT2 display a similar lamellar stacking distance (*d*_100_ ∼ 1.92 nm), which is shorter than for PNDIT2 (*d*_100_ ∼ 2.5 nm): we propose an explanation later on in the paragraph. Furthermore, the presence of a second-order diffraction of the lamellar stacking in the E-PNDIT2 pattern (*Q* ∼ 6.50 nm^–1^) implies a high crystallinity. The (001) scattering peak, which corresponds to the chain backbone repeat length, is not detectable in the DMP-PNDIT2 and E-PNDIT2 diffractograms. However, the chain backbone repeat length is not expected to vary with the addition of the dimethoxyphenyl groups, or of the encapsulating rings, and can be assumed equal to 1.37 nm as for PNDIT2. The π-stacking distance has a large variability, hence disorder, for all polymers, as indicated by the wide diffraction at high *Q*-values. For E-PNDIT2 and DMP-PNDIT2, the average d-spacing corresponding to the π-stacking distance (*d*_010_ ∼ 0.45 nm) is longer than for PNDIT2 (*d*_010_ ∼ 0.39 nm). This is a clear consequence of the steric hindrance provided by the dimethoxyphenyl groups in DMP-PNDIT2 and by the encapsulating rings in E-PNDIT2. Remarkably, considering the axial symmetry of the encapsulating ring with a diameter of about 1 nm, it follows from simple geometrical considerations that a π-stacking distance of about 0.45 nm between the NDI moieties implies a close interpenetration of the encapsulating rings, as schematized in Figure 3f. This is further corroborated by the appearance of three sharp in-plane diffractions (*Q* = 7.46, 9.22, 13.30 nm^–1^), which do not find correspondence in the PNDIT2 diffractogram, and are tentatively assigned to interlocking of the encapsulating rings of adjacent polymer chains. Overall, molecular encapsulation promotes a significant thin film crystallinity, induces a preferential edge-on orientation, and controls the packing of the E-PNDIT2 chains. On the one hand, it regulates the interchain distances in the polymer aggregates, making the lamellar stacking distance shorter and the π-stacking distance longer than in PNDIT2. On the other hand, the interlocking rings support a planarization of the polymer backbones with respect to unaggregated chains, which is also confirmed by the rigid red shift of the absorption spectrum in thin film with respect to solution. Interestingly, an increased structural order of E-PNDIT2 is achieved upon thermal annealing. Indeed, nonannealed films show GIWAXS patterns exhibiting a few, low-intensity, and diffuse diffractions, suggesting a low degree of structural order, in contrast with the sharper diffraction of samples annealed at 180 °C that we described in Figure 3. GIWAXS measurements performed in situ during annealing of the E-PNDIT2 film provide insights into the development of such a microstructure (Figures S11 and S12). The low ordered microstructure is kinetically quenched during spin-casting. Upon heating the as-cast film at temperatures >150 °C, its microstructure undergoes a crystallization, achieving a higher degree of structural order. Further annealing at higher temperatures (up to 300 °C) does not lead to other structural changes.

**Table 2:** Main Diffraction Peaks and Corresponding *d*-Spacing for PNDIT2, DMP-PNDIT2, and E-PNDIT2^a^

	(100) lamellar stacking		(001) backbone chain repeat		(010) π-stacking	
	*Q* (nm^–1^)	*d*_100_ (nm)	*Q* (nm^–1^)	*d*_001_ (nm)	*Q* (nm^–1^)	*d*_010_ (nm)
PNDIT2	2.55 (IP)	2.46 (IP)	4.57 (IP)	1.37 (IP)	15.8 (OOP)	0.39 (OOP)
DMP-PNDIT2	3.22 (IP)	1.95 (IP)	NA	NA	14.1 (IP)	0.45 (IP)
	3.34 (OOP)	1.88 (OOP)			14.6 (OOP)	0.43 (OOP)
E-PNDIT2	3.26 (OOP)	1.92 (OOP)	NA	NA	14.0 (IP)	0.45 (IP)

a The diffractions occurring predominantly in the in-plane (IP) and in the out-of-plane (OOP) directions are indicated within parentheses. Since there is not a preferential alignment detected in the DMP-PNDIT2 film, the values for both the in-plane and out-of-plane diffractions are reported.

### 2.4 Charge Transport

**Figure 4:**
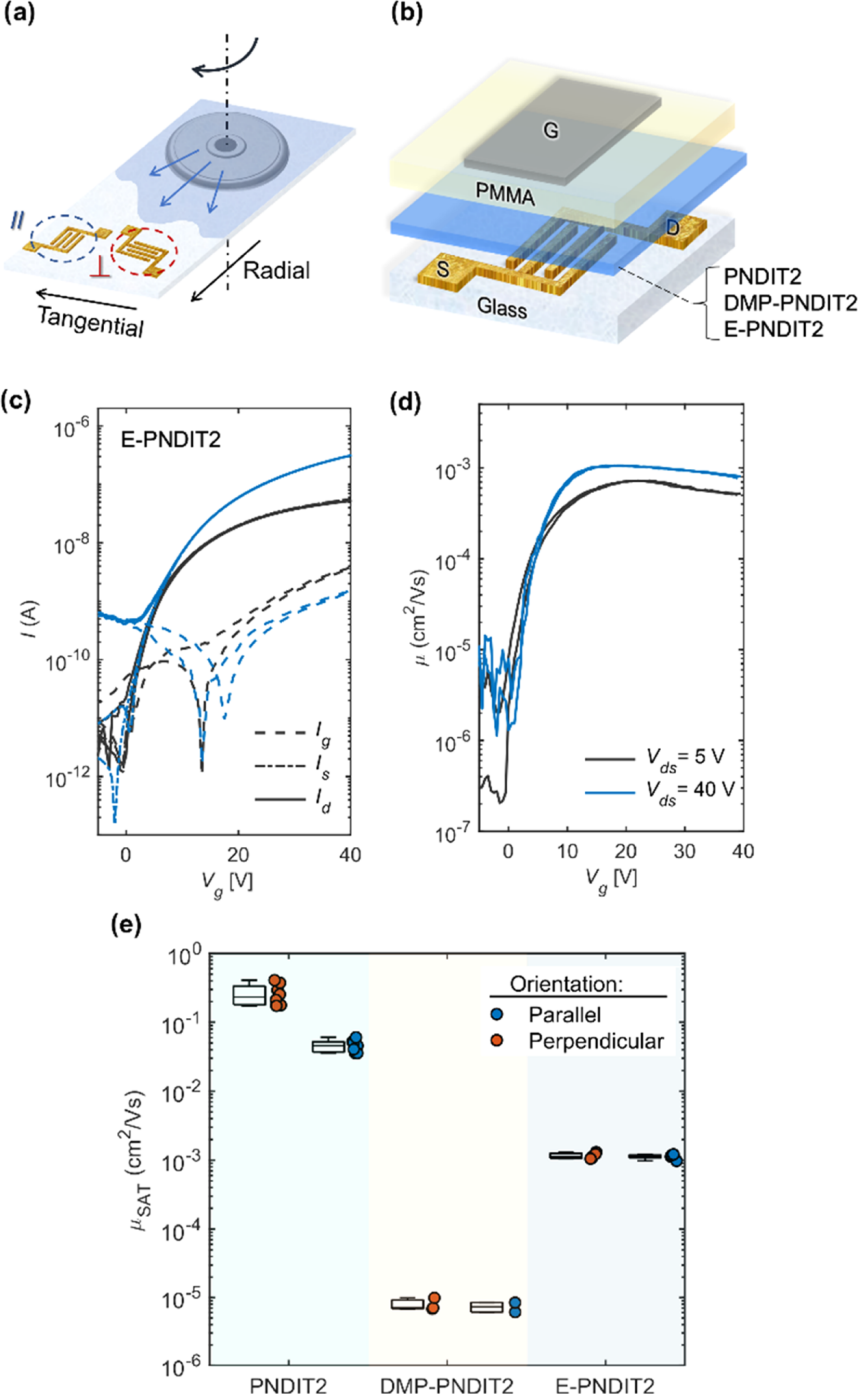
(a) Scheme of the deposition of the semiconductor layer (PNDIT2, DMP-PNDIT2, and E-PNDIT2) by off-center spin-coating. Glass substrates are prepatterned with gold interdigitated source and drain contacts, oriented both perpendicularly (⊥) and parallelly (*∥*) to the alignment direction. (b) TGBC transistor structure composed of a glass substrate, a gold source, drain contacts, a semiconducting film, a PMMA dielectric layer, and an aluminum gate electrode. (c) Transfer curves of an optimized device with the E-PNDIT2 active layer in linear and saturation regimes (in black and blue, respectively) and (d) plot of the related gate voltage-dependent mobility. The device has a channel length *L* = 10 μm and a channel width *W* = 2 mm. (e) Box-plot comparing the mobility values in the saturation regime (extracted from the linear fit of *I*_ds_^0.5^ in the range *V*_g_ = 20–30 V; details are provided in Figure S19) of transistors based on PNDIT2, DMP-PNDIT2, and E-PNDIT2. The values are grouped accordingly to the parallel/perpendicular orientation of the contacts.

To investigate the effect of molecular encapsulation on charge transport, we fabricated organic field-effect transistors (OFETs) in a top-gate bottom-contact (TGBC) architecture, employing PNDIT2, DMP-PNDIT2, and E-PNDIT2 as the active layer. Source and drain interdigitated contacts were defined by photolithography on glass substrates, followed by a thermal evaporation of 30 nm of gold with a 2 nm chromium adhesion layer. The semiconducting layers, either PNDIT2, DMP-PNDIT2, or E-PNDIT2, were deposited by off-center spin-coating with the same conditions adopted for the preparation of the films investigated by GIWAXS. As a result of the applied centrifugal force, this deposition technique induces a preferential alignment of PNDIT2 microfibrils and a corresponding charge transport anisotropy.^56,65^ It is therefore interesting to evaluate if any transport anisotropy can derive from aligned structures also for DMP-PNDIT2 and E-PNDIT2. To this extent, contacts with both parallel and perpendicular orientations with respect to the radial spinning direction were employed to assess the eventual charge transport anisotropy typical of aligned textures (Figure 4a). A 500 nm film of poly(methyl methacrylate) (PMMA) was spin-coated to obtain a dielectric layer, which notoriously provides an interface with PNDIT2 suitable for charge transport.^66,67^ Finally, aluminum gate electrodes were thermally evaporated to complete the TGBC transistor structure (Figure 4b).

To compare the electrical transport properties of the three polymers in films with optimized morphology, we performed preliminary tests using different solvents and deposition parameters. The UV–vis absorption spectra of films of E-PNDIT2 deposited from various organic solvents at a concentration of 5 g/L are shown in Figure S13. No differences are observed in the normalized absorption spectra, implying identical electronic transitions in thin film and thus a common intramolecular structure and intermolecular arrangement of the polymer chains. Instead, the variation of the absolute absorbance is ascribed to a different thickness and coverage of the films over the 2 × 2 cm^2^ substrate. The films deposited from toluene displayed the best uniformity. Moreover, toluene is one of the most employed solvents to deposit films of PNDIT2 with high charge transport performance due to the property of promoting a high level of preaggregation in solution.^55,57^ Hence, we compare here transistors with the three polymers deposited by off-center spin-coating from a 5 g/L toluene solution. The films were annealed for 30 min at 180 °C immediately after the deposition, which has a significant impact on the microstructure of E-PNDIT2, as confirmed by in situ GIWAXS measurements (Figures S11 and S12), UV–vis spectroscopy (Figure S14), and field-effect mobility evaluation (Figure S15). Figure 4c displays the transfer curves in the linear (*V*_ds_ = 5 V, in black) and saturation regimes (*V*_ds_ = 40 V, in blue) of a representative device based on E-PNDIT2. The encapsulated polymer shows a clear n-type field-effect behavior, with an *I*_on_/*I*_off_ ∼ 10^4^ and a field-effect mobility μ ∼ 10^–3^ cm^2^ V^-1^ s^-1^, which is weakly dependent on the gate voltage above threshold (Figure 4d). No differences in charge transport performance were observed in transistors with contacts oriented parallel or perpendicularly to the centrifugal force induced by off-center spin-coating. As opposed to PNDIT2 (Figure S16), the films do not show anisotropic charge transport. Such evidence suggests the absence of oriented supramolecular assemblies for E-PNDIT2. The surface of E-PNDIT2, imaged by atomic force microscopy (AFM) (Figure S17), consistently presents a subnanometric roughness, and no structuration of the polymer fibrils typically observed in PNDIT2 films can be detected within the resolution of the instrument. The transfer characteristics for representative devices of PNDIT2, DMP-PNDIT2, and E-PNDIT2 are shown in Figure S18, along with the plots of the mobility as a function of the gate voltage.

Figure 4e collects the distribution of field-effect mobility in the saturation regime from devices based on the three polymers grouped depending on the orientation of the contacts. The three polymers present significantly different field-effect mobilities. DMP-PNDIT2 provides the worst charge transport performance, with isotropic and very poor field-effect mobility on the order of 10^–5^ cm^2^ V^-1^ s^-1^. According to the previous literature, PNDIT2 shows the highest field-effect mobility and a clear charge transport anisotropy due to the fibrillar alignment induced by off-center spin-coating, which is fundamental to form effective charge percolation paths.^55,59^ Transistors with contacts oriented perpendicularly to the alignment direction offer charge mobility values >10^–1^ cm^2^ V^–1^ s^–1^. The performance of the transistors based on E-PNDIT2 stands halfway between those of PNDIT2 and DMP-PNDIT2, with an isotropic mobility of about 10^–3^ cm^2^ V^–1^ s^–1^. Importantly, molecular encapsulation still allows π-stacking between different polymer backbones but with a longer *d*-spacing than in PNDIT2, which is likely associated with a reduced charge-transfer rate. Together with the absence of a directional supramolecular order, as found by AFM, such structural considerations explain the reduced charge carrier mobility in E-PNDIT2 with respect to PNDIT2.

## 3 Conclusions

This article reports the synthesis of naphthalene diimide bithiophene-based conjugated polymers and studies the effect of molecular encapsulation (around the bithiophene unit) on the photophysical and charge transport properties of the polymer. The molecular encapsulation prevents preaggregation of the polymer chains in all employed organic solvents (effect on the intrachain packing, i.e., coiling). The electronic structure of the core polymer is mostly preserved, with a positive effect on the backbone conjugation ascribed to increased planarization of the polymer backbone. The encapsulating rings influence the solid-state packing, changing the interchain distances and the molecular orientation in thin films. Semicrystalline order is likely achieved through interdigitating macrocycles. Remarkably, even though the associated *d*-spacing for E-PNDIT2 is longer than for PNDIT2, the π-stacking between NDI moieties is preserved, affording a decent field-effect mobility of ∼ 10^–3^ cm^2^ V^–1^ s^–1^. Therefore, molecular encapsulation emerges as a general approach to tune the nanomorphology of casted films, without altering the electronic structure of the core polymer and still allowing charge transport properties in thin films. Supramolecular fibrillary microstructures, which are essential to form effective percolative paths for charge transport, are not formed in the absence of aggregated chains in solution. Still, avoiding preaggregation in solution holds promise for improving the intermixing of multiple components in a common solvent. This feature is strongly desirable in blends of donor and acceptor organic semiconductors for bulk heterojunction solar cells and to reach effective intercalation of molecular dopants within a polymeric matrix for organic thermoelectrics.

## 4 Experimental Section

### 4.1 Synthesis

A detailed description of the synthetic process of the naphthalene diimide bithiophene copolymers, and of their characterization, is reported in the Supporting Information.

### 4.2 Thin Film Deposition

PNDIT2, DMP-PNDIT2, and E-PNDIT2 were dissolved in toluene (Sigma-Aldrich) at a concentration of 5 g/L unless stated otherwise in the text. The thin films of the three polymers were deposited in a nitrogen glovebox by off-center spin-coating onto glass substrates (low alkali 1737F Corning glasses, purchased from Präzisions Glas & Optik GmbH). A double-step process was adopted (first step: 500 rpm/s for 10 s with acceleration 50 rpm/s; second step: 1000 rpm/s for 60 s with acceleration 2000 rpm/s). The deposition was followed by annealing at 180 °C for 30 min. The obtained films had a thickness of 40 ± 10 nm.

### 4.3 Optical Measurements

The absorption spectra were measured with a PerkinElmer Lambda 1050 UV/vis/near-infrared (NIR) spectrometer. The photoluminescence (PL) spectra and the excitation profiles were collected with a iHR320Horiba NanoLog Fluorometer. All absorption and PL spectra were collected in air and at room temperature unless stated otherwise.

### 4.4 Film Topography and Thickness

The film thickness was measured with a KLA Tencor P-17 Surface Profiler. The surface topography of the E-PNDIT2 thin film was imaged with an Agilent 5500 atomic force microscope operating in a tapping mode.

### 4.5 Grazing-Incidence Wide-Angle X-ray Scattering (GIWAXS)

GIWAXS measurements were performed at the noncrystalline diffraction beamline (BL11-NCD-Sweet) at ALBA Synchrotron Radiation Facility in Barcelona (Spain). A detector (Rayonix, WAXS LX255-HS) with a resolution of 1920 × 5760 pixels was used to collect the scattering signals. The sample holder position was calibrated with the chromium oxide (Cr_2_O_3_) standard. The incident energy was 12.4 eV, and the sample-to-detector distance was set at 216.5 mm. The angle of incidence α_i_ was 0.12°, and the exposure time was 1 s. 2D-GIWAXS patterns were corrected as a function of the components of the scattering vector with a MATLAB script developed by Aurora Nogales and Edgar Gutiérrez.^68^ Thin films were cast onto highly doped silicon substrates following the same processing route used for the device fabrication.

### 4.6 OFET Fabrication and Electrical Characterization

The OFETs were fabricated with a bottom-contact top-gate architecture onto glass substrates (low alkali 1737F Corning glasses). Bottom source and drain interdigitated electrodes were defined by standard photolithography and deposited by thermal evaporation of a 30 nm thick Au layer with a 3 nm thick Cr adhesion layer. The semiconducting layer (either PNDIT2, DMP-PNDIT2, or E-PNDIT2) was deposited by off-center spin-coating in a nitrogen glovebox according to the procedure described above. Successively, a 500 nm thick dielectric layer of PMMA (*M*_w_ = 120 000, purchased from Sigma-Aldrich, dissolved in *n*-butyl acetate at a concentration of 80 g/L) was deposited by on-center spin-coating (1300 rpm for 60 s with 1000 rpm/s acceleration) and then baked at 80 °C for ∼1 h. Finally, the top-gate electrode was obtained by thermal evaporation of a 40 nm Al layer patterned using a shadow mask. OFET transfer and output electrical characteristics were measured with a semiconductor parameter analyzer (Agilent B1500A) in a nitrogen glovebox on a Wentworth Laboratories probe station. The samples were further annealed at 120 °C overnight before measuring the electrical characteristics. The mobility was extracted from the linear fit of *I*_ds_^0.5^ from the transfer curves in the saturation regime (in the range *V*_g_ = 20–30 V; details are provided in Figure S19).

## Supporting Information Available

Detailed information on the synthesis of all compounds, NMR spectra, DFT-computed dihedral angles and HOMO/LUMO levels, and additional experimental details (PDF)
