# Supplementary material for: Effects of Molecular Encapsulation on the Photophysical and Charge Transport Properties of a Naphthalene Diimide Bithiophene Copolymer
Source: Chem Mater. 2022 Sep 5;34(18):8324–35. doi: 10.1021/acs.chemmater.2c01894 (PMC9520976; doi:10.1021/acs.chemmater.2c01894)
Supplement: Supplementary file 1 — cm2c01894_si_001.pdf [file cm2c01894_si_001.pdf]

Supporting Information

Effects of Molecular Encapsulation on the Photophysical and Charge Transport Properties of a Naphthalene Diimide Bithiophene Copolymer

Stefano Pecorario[‡,1,2], Jeroen Royakkers[‡,3,4,5], Alberto D. Scaccabarozzi[1], Francesca Pallini[6], Luca Beverina[6], Hugo Bronstein[*,4,5], Mario Caironi[*,1]

[1] Center for Nano Science and Technology@PoliMi, Istituto Italiano di Tecnologia, via Giovanni Pascoli 70/3, Milan 20133, Italy

[2] Department of Energy, Micro and Nanostructured Materials Laboratory - NanoLab, Politecnico di Milano, Via Ponzio 34/3, Milano, 20133, Italy.

[3] Sensor Engineering Department, Faculty of Science and Engineering, Maastricht University, P.O. Box 616, 6200 MD Maastricht, The Netherlands

[4] Department of Chemistry, University of Cambridge, Lensfield Road, Cambridge, CB2 1EW UK

[5] Cavendish Laboratory, University of Cambridge, Cambridge, CB3 0HE UK

[6] Department of Materials Science, Università di Milano-Bicocca, via Cozzi 55, 20125 Milan, Italy

[‡] These authors contributed equally

* Corresponding authors

Table of Contents:

1) Synthesis
    - General Experimental Information 3
    - Monomers synthesis 4
    - Polymers synthesis 7
    - NMR Spectra 9
2) DFT Calculations
    - Dihedral Angles 17
    - HOMO/LUMO levels 19
3) Thermal Characterization
    - Figure S1 22
    - Figure S2 24
4) Optical Properties
    - Figure S3 25
    - Figure S4 25
    - Figure S5 26
    - Figure S6 26
    - Figure S7 27
    - Figure S8 27
    - Figure S9 28
5) Thin Film Microstructure
    - Figure S10 28
    - Figure S11 29
    - Figure S12 30
6) Charge Transport
    - Figure S13 31
    - Figure S14 31
    - Figure S15 32
    - Figure S16 33
    - Figure S17 34
    - Figure S18 34
    - Figure S19 35

References 36

1) Synthesis

- **General Experimental Information**

All reactions were performed in oven-dried glassware under argon atmosphere and magnetic stirring, unless stated otherwise. The reactions were protected from light using aluminum foil. Chemicals and solvents were purchased from chemical suppliers (Sigma-Aldrich, TCI, Fluorochem, Acros Organics, Alfa Aesar, SLS, Fisher Scientific) and used as received unless stated otherwise. The Br_2-NDI-2OD co-monomer was prepared according to our previous literature procedure.[1] Reactions were monitored through thin layer chromatography (TLC) using DC Fertigfolien ALUGRAM aluminium sheets coated with silica gel. Column chromatography was carried out on a Biotage® Isolera™ Four with Biotage® SNAP or Sfar cartridges (10 g, 20 g, 50 g or 100 g). ^{1}H NMR spectra were recorded on a 400 MHz Avance III HD Spectrometer, 400 MHz Neo Prodigy Spectrometer, 400 MHz Smart Probe Spectrometer or a 500 MHz DCH Cryoprobe Spectrometer in the stated solvent using residual protic solvent $CHCl_3$ (δ = 7.26 ppm, s) or DMSO (δ = 2.50 ppm, s) as the internal standard. ^{1}H NMR chemical shifts are reported to the nearest 0.01 ppm. ^{13}C NMR spectra were recorded on the 500 MHz DCH Cryoprobe Spectrometer in the stated solvent using the residual protic solvent $CHCl_3$ (δ= 77.16 ppm, t) or DMSO (δ = 39.52 ppm, s) as the internal standard. ^{13}C NMR chemical shifts are reported to the nearest 0.01 ppm. Mass spectra were obtained using a Waters LCT Premier, Waters Vion IMS Qtof, Finnigan MAT 900XP or Waters MALDI micro MX spectrometer at the Department of Chemistry, University of Cambridge. The number-average (Mn) and weight-average (Mw) molecular weights were determined against a polystyrene standard using an Agilent Technologies 1200 Series GPC-SEC System in chlorobenzene at 80 °C.

- **Monomers Synthesis**

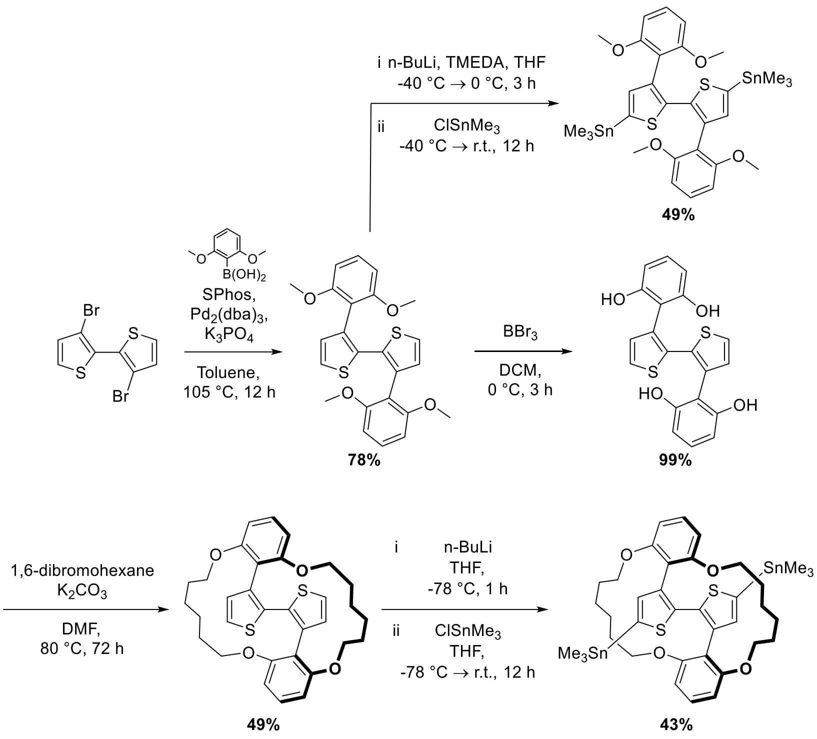

3,3'-bis(2,6-dimethoxyphenyl)-2,2'-bithiophene (DMP-BT)[2]

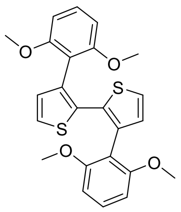

Under argon, a solution of 3,3'-dibromo-2,2'-bithiophene (2.2321 g, 6.8879 mmol), 2,6-dimethoxyphenylboronic acid (5.0 g, 27.4755 mmol), SPhos (283.5 mg, 0.6906 mmol), $Pd_2(dba)_3$ (198.7 mg, 0.2170 mmol) and K_3PO_4 (7.0982 g, 33.4395 mmol) in toluene (25 mL) and water (5 mL) was stirred at 105°C for 12 h. The reaction mixture was cooled to room temperature, washed with water and extracted with toluene (1x). The organic phase was concentrated *in vacuo* and the residue was sonicated in methanol. The resulting white solids were collected by filtration. Next, the product was further purified using silica column chromatography (loaded with 100% hexane, eluted with 50% DCM). The product fractions were concentrated *in vacuo* to afford the product as a white solid (2.3491 g, 5.3564 mmol, 78%).

^{1}H NMR (400 MHz, CDCl3) δ 7.14 (d, J = 5.2 Hz, 1H), 7.10 (t, J = 8.3 Hz, 1H), 6.87 (d, J = 5.2 Hz, 1H), 6.36 (d, J = 8.3 Hz, 2H), 3.50 (s, 6H). **HRMS** (ASAP-TOF): Calculated for $C_{24}H_{23}O_4S_2^+$: 439.1038. Found *m/z* 439.1029 $[M+H]^+$.

2,2’-Bis(trimethylstannyl)-3,3'-bis(2,6-dimethoxyphenyl)-2,2'-bithiophene (DMP-BT-$SnMe_3$)

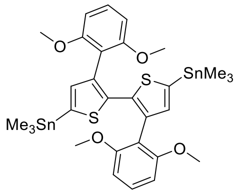

Under argon, n-BuLi (3.84 mL, 1.6 M, 6.1440 mmol) was added dropwise at -40 °C to a solution of DMP-BT (1.0 g, 2.2802 mmol) in dry THF (100 mL) and TMEDA (0.95 mL). The reaction was stirred at this temperature for 5 minutes, after which it was warmed to 0 °C and left stirring for 3 h. Next, it was cooled back to -40 °C and trimethyltinchloride solution (7 mL, 1.0 M in THF, 7.0 mmol) was added. The reaction was left to warm to room temperature and it was stirred for 12 h. Next, water (~50 mL) was added and the organic layer was separated. The aqueous layer was then extracted with DCM (2 x) and the combined organic layer was dried over $MgSO_4$, filtered and concentrated *in vacuo*. The resulting solid was sonicated in methanol and collected by filtration to afford 1.4 g of crude material. The crude was recrystallized twice from acetonitrile (First recrystallization: dissolve crude in 250 mL ACN, add a spoon of charcoal, filter through celite, reduce volume to ~100 mL and let it cool down. The same procedure was followed for the second recrystallization, but this time it was reduced to 50-60 mL). The desired product was obtained as a white crystalline solid (854.3 mg, 1.1179 mmol, 49%).

^{1}H NMR (700 MHz, CD_2Cl_2) δ 7.09 (t, *J* = 8.3 Hz, 2H), 6.94 – 6.83 (m, 2H), 6.36 (d, *J* = 8.3 Hz, 4H), 3.47 (s, 12H), 0.46 – 0.16 (s+d, 18H).**^{13}C NMR** (176 MHz, CD_2Cl_2) δ 158.37, 140.63, 140.22, 134.68, 131.82, 128.62, 114.78, 104.52, 55.63, -8.05. **HRMS** (ASAP-TOF): Calculated for $C_{30}H_{39}O_4S_2Sn_2^+$: 765.0323. Found *m*/*z*: 765.0289 $[M]^+$.

3,3'-bis(2,6-dihydroxyphenyl)-2,2'-bithiophene (DHP-BT)[2]

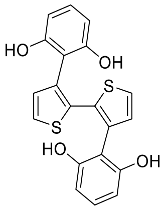

Under argon, a 1M BBr_3 solution (in DCM, 27.4 mL, 27.4 mmol) was added dropwise at – 10 °C to a solution of 3,3'-bis(2,6-dimethoxyphenyl)-2,2'-bithiophene (3.0 g, 6.8406 mmol) in DCM (29 mL). The reaction was stirred for 6 h at – 10 °C. Next, the reaction mixture was poured into 100 mL of 1M $NaHCO_3$ solution and the white solids were collected by filtration. The solids were re-dissolved in EtOAc, dried over $MgSO_4$ and concentrated *in vacuo*. The solids were then sonicated in DCM (2x) and collected by filtration to afford the product as a white solid (2.61 g, 6.8245 mmol, >99%).

^{1}**H NMR** (400 MHz, DMSO) δ 8.86 (s, 4H), 7.27 (d, *J* = 5.1 Hz, 2H), 6.97 (t, *J* = 8.1 Hz, 2H), 6.83 (d, *J* = 5.1 Hz, 2H), 6.36 (d, *J* = 8.1 Hz, 4H). **HRMS** (ASAP-TOF): Calculated for $C_{20}H_{15}O_4S_2^+$: 383.0412. Found *m*/*z* 383.0410 [M+H]$^+$.

Encapsulated Bithiophene (E-BT)[2]

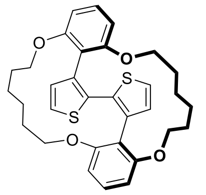

Under argon, 3,3'-bis(2,6-dihydroxyphenyl)-2,2'-bithiophene (1.0 g, 2.6147 mmol), K_2CO_3 (2.4213 g, 17.5190 mmol) and dry DMF (300 mL) were added to a 500 mL round-bottomed flask and it was heated at 50 °C for 1 h. To this, a solution of 1,6-dibromohexane (0.8044 mL, 5.2294 mmol) in dry DMF (300 mL) was added dropwise over 1 h. The mixture was heated to 80 °C and stirred for 48 h. The reaction mixture was concentrated *in vacuo*, re-dissolved in the minimal amount of chloroform and hexane and purified by silica column chromatography, using 50-70% chloroform in hexane. The product fractions were concentrated *in vacuo*, sonicated in methanol, collected by filtration and dried under high vacuum to afford the product as a white solid (704.3 mg, 1.2882 mmol, 49%).

^{1}**H NMR** (400 MHz, CDCl3) δ 7.38 (t, J = 8.3 Hz, 2H), 6.98 (d, J = 5.1 Hz, 2H), 6.75 (d, J = 5.1 Hz, 2H), 6.69 (d, J = 8.3 Hz, 4H), 4.12 – 3.97 (m, 4H), 3.75 (ddd, J = 9.1, 7.7, 5.1 Hz, 4H), 1.52 – 1.39 (m, 8H), 1.26 – 1.07 (m, 4H), 0.93 (ddd, J = 14.2, 12.2, 6.9 Hz, 4H). ^{13}**C NMR** (176 MHz, $CDCl_3$) δ 159.10, 133.51, 131.20, 130.16, 129.52, 122.73, 117.62, 107.26, 69.90, 30.43, 27.40. **HRMS** (ASAP-TOF): Calculated for $C_{32}H_{35}O_4S_2^+$: 547.1977. Found *m*/*z* 547.1967 [M+H]$^+$.

Encapsulated Bithiophene Monomer (E-BT-SnMe$_3$)

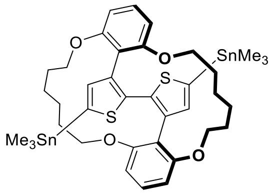

Under argon, n-BuLi (0.85 mL, 1.6 M, 1.3632 mmol) was added dropwise at -40 °C to a solution of E-BT (300 mg, 0.6214 mmol) in dry THF (5 mL) and TMEDA (0.26 mL). The reaction was stirred at this temperature for 5 minutes, after which it was warmed to 0 °C and left stirring for 3 h. Next, it was cooled back to -40 °C and trimethyltinchloride solution (1.5 mL, 1.0 M in THF, 1.50 mmol) was added. The reaction was left to warm to room temperature and it was stirred for 12 h. Next, water (~50 mL) was added and the organic layer was separated. The aqueous layer was then extracted with DCM (2 x) and the combined organic layer was dried over $MgSO_4$, filtered and concentrated *in vacuo*. The resulting

solid was sonicated in methanol and collected by filtration. The crude was recrystallized from acetonitrile (and a small amount of $CHCl_3$) to afford the desired product as a white crystalline solid (235.7 mg, 0.2702 mmol, 43%).

^{1}H NMR (700 MHz, $CDCl_3$) δ 7.34 (t, *J* = 8.3 Hz, 2H), 6.80 – 6.74 (s, 2H), 6.68 (d, *J* = 8.3 Hz, 4H), 4.00 (dt, *J* = 8.7, 4.2 Hz, 4H), 3.69 (td, *J* = 9.4, 2.9 Hz, 4H), 1.49 (d, *J* = 10.3 Hz, 4H), 1.42 (dd, *J* = 11.8, 8.2 Hz, 4H), 1.09 (dd, *J* = 17.0, 6.9 Hz, 4H), 0.91 – 0.84 (m, 4H), 0.35 – 0.15 (m, 18H). **^{13}C NMR** (176 MHz, CD_2Cl_2) δ 159.49, 140.16, 138.36, 134.21, 132.88, 130.23, 118.48, 107.90, 70.37, 30.68, 27.64, -8.18. **HRMS** (ASAP-TOF): Calculated for $C_{38}H_{50}O_4S_2Sn_2^+$: 872.1189 Found *m/z* 872.1221 $[M]^+$.

- **Polymers Synthesis**

PNDIT2[3]

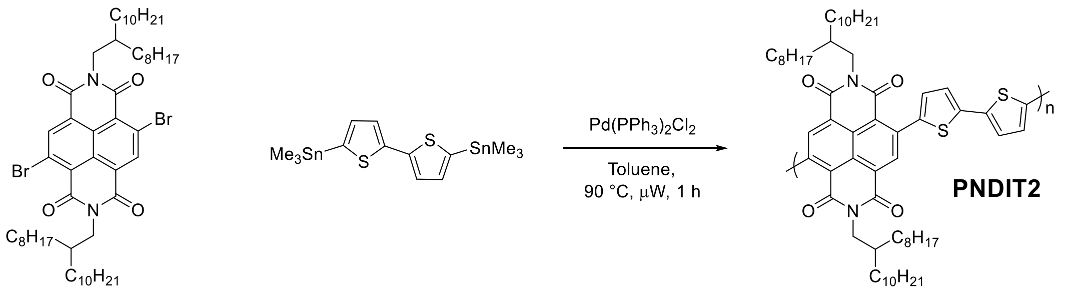

Under argon, a mixture of Br_2-NDI-2OD (120 mg, 0.1218 mmol), 5,5'-bis(trimethylstannyl)-2,2'-bithiophene (60.6 mg, 0.1218 mmol), $Pd(PPh_3)_2Cl_2$ (4.5 mg, 0.0064 mmol) and anhydrous toluene (3 mL) was stirred at 90 °C under microwave irradiation for 1 h. The resulting blue viscous liquid was precipitated into stirring methanol (~150 mL) and the polymer was collected by filtration and subjected to soxhlet extraction in acetone, hexane and $CHCl_3$. The $CHCl_3$ fraction was concentrated *in vacuo*, precipited in methanol (~150 mL), collected by filtration and dried under high vacuum to afford the polymer as a blue-green plastic (118.5 mg, 0.1195 mmol, 98%); Mn = 45.0 kDa, Mw = 136.1 kDa, PDI = 3.02.

DMP-PNDIT2

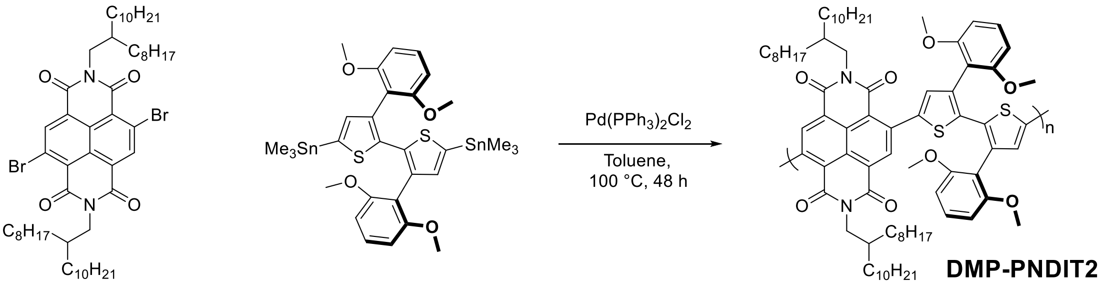

Under argon, a mixture of Br_2-NDI-2OD (150 mg, 0.1523 mmol), DMP-BT-$SnMe_3$ (116.4 mg, 0.1523 mmol), $Pd(PPh_3)_2Cl_2$ (5.57 mg, 0.0079 mmol) and anhydrous toluene (4 mL) was stirred at 100 °C for

48 h. The resulting blue-green liquid was precipitated into stirring methanol (~150 mL) and the polymer was collected by filtration and subjected to soxhlet extraction in acetone, hexane and $CHCl_3$. The $CHCl_3$ fraction was concentrated *in vacuo*, precipited in methanol (~150 mL), collected by filtration and dried under high vacuum to afford the polymer as a blue-green solid (215.4 mg, 0.1431 mmol, 94%); Mn = 19.4 kDa, Mw = 37.1 kDa, PDI = 1.91.

E-PNDIT2

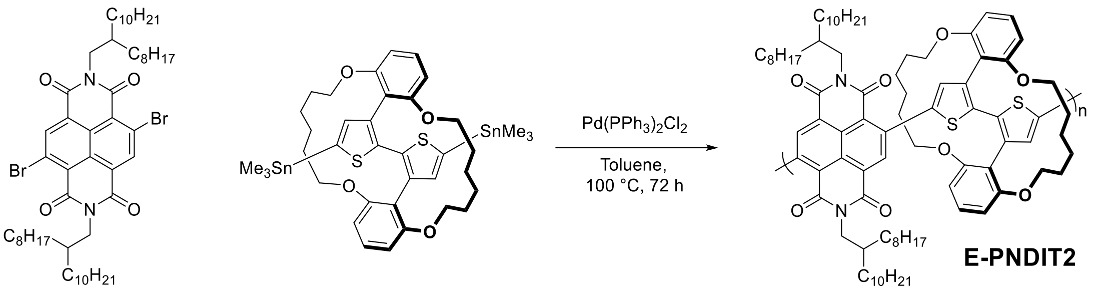

Under argon, a mixture of Br_2-NDI-2OD (70 mg, 0.0711 mmol), E-BT-$SnMe_3$ (62 mg, 0.0711 mmol), $Pd(PPh_3)_2Cl_2$ (2.6 mg, 0.0037 mmol) and anhydrous toluene (1.7 mL) was stirred at 90 °C for 72 h. The resulting blue-green liquid was precipitated into stirring methanol (~150 mL) and the polymer was collected by filtration and subjected to soxhlet extraction in acetone, hexane and $CHCl_3$. The $CHCl_3$ fraction was concentrated *in vacuo*, precipited in methanol (~150 mL), collected by filtration and dried under high vacuum to afford the polymer as a blue-green solid (59.9 mg, 0.0437 mmol, 61%); Mn = 19.9 kDa, Mw = 46.4 kDa, PDI = 2.33.

- **NMR Spectra**

3,3'-bis(2,6-dimethoxyphenyl)-2,2'-bithiophene (DMP-BT)[2]

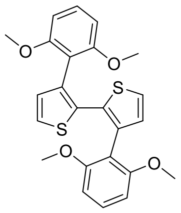

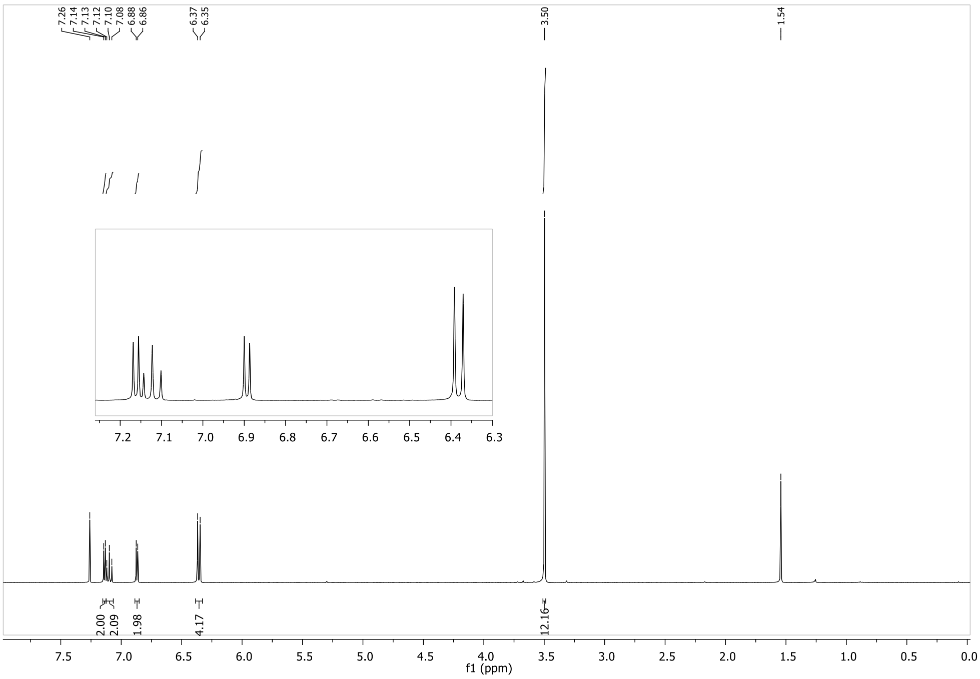

2,2’-Bis(trimethylstannyl)-3,3'-bis(2,6-dimethoxyphenyl)-2,2'-bithiophene (DMP-BT-$SnMe_3$)

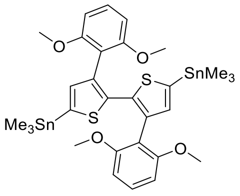

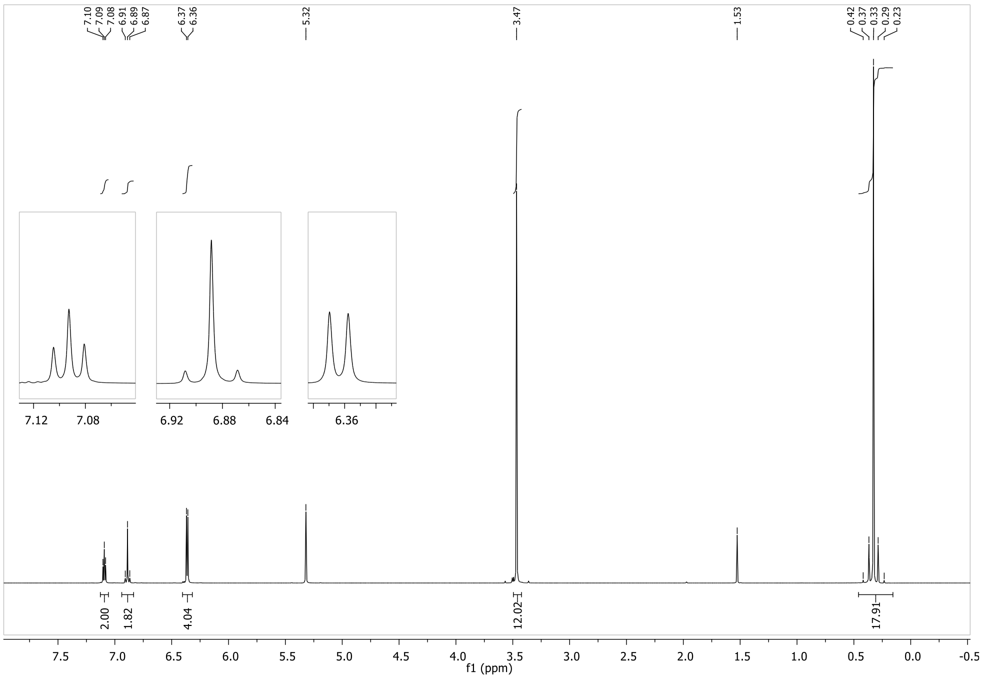

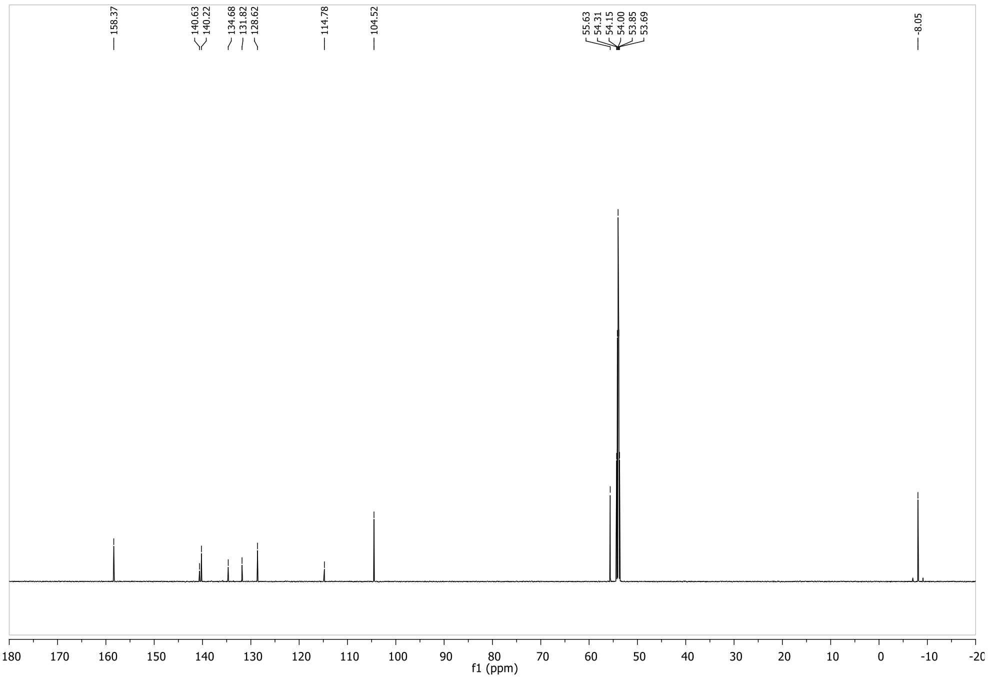

3,3'-bis(2,6-dihydroxyphenyl)-2,2'-bithiophene (DHP-BT)[2]

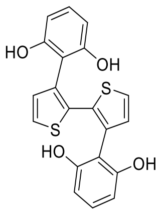

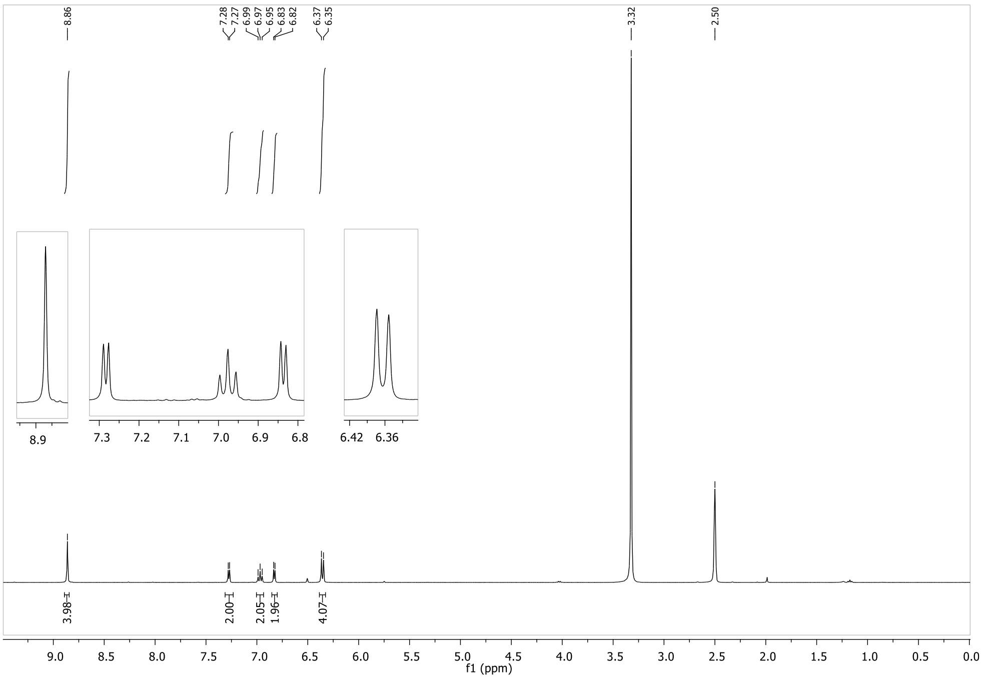

Encapsulated Bithiophene (E-BT)[2]

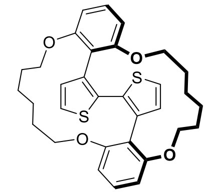

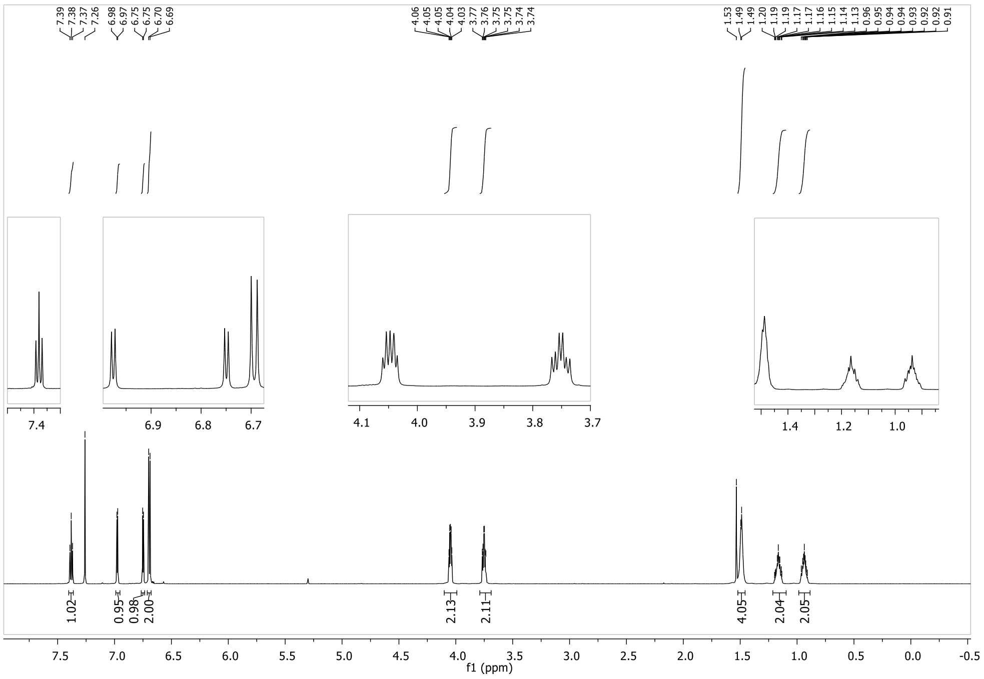

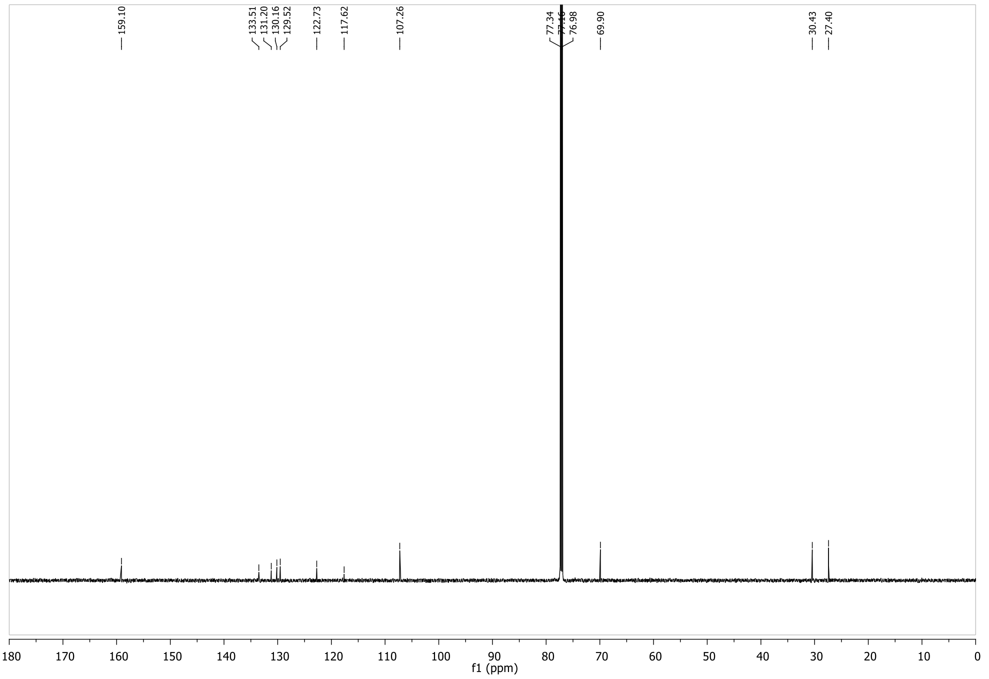

Encapsulated Bithiophene Monomer (E-BT-SnMe$_3$)

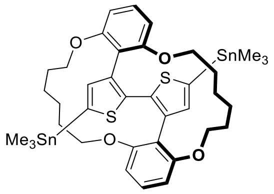

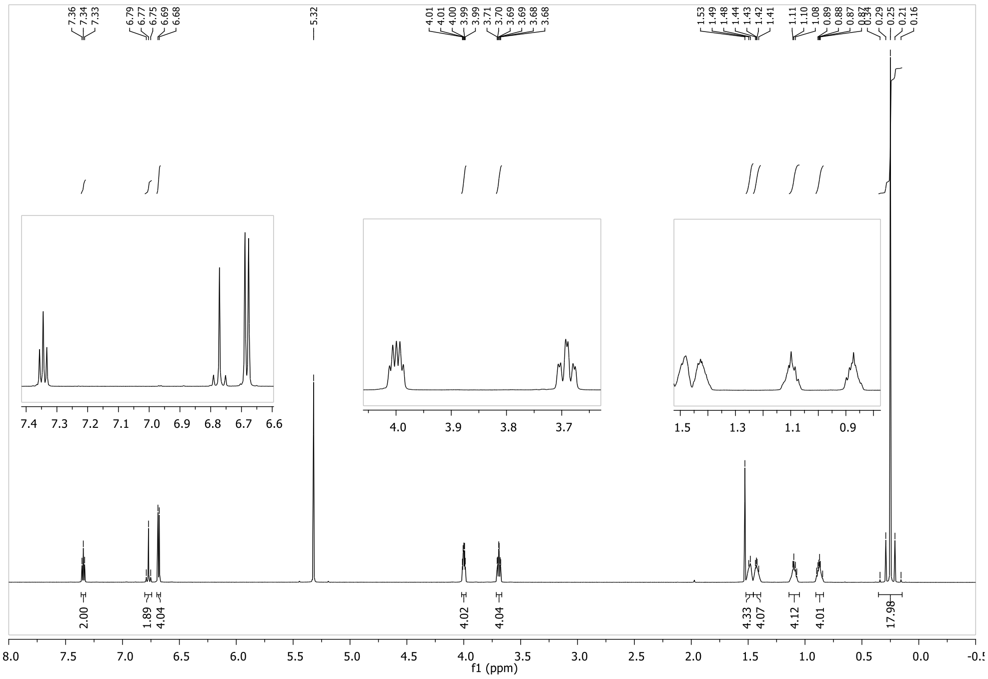

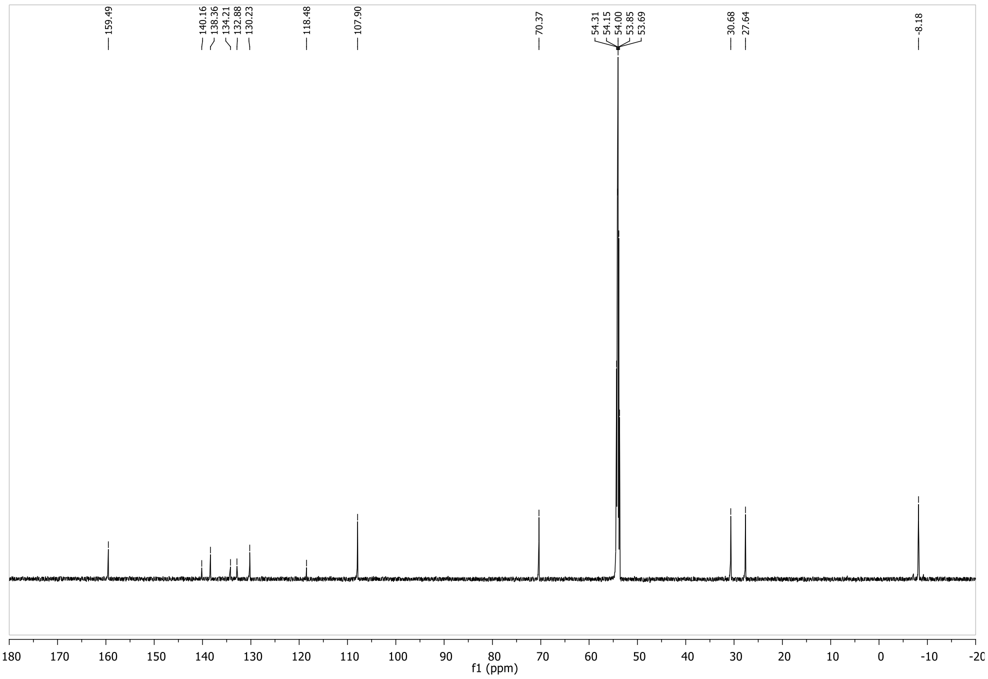

PNDIT2

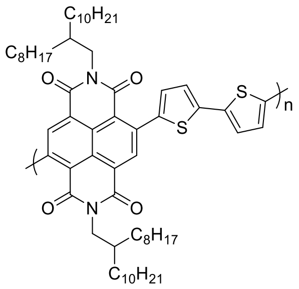

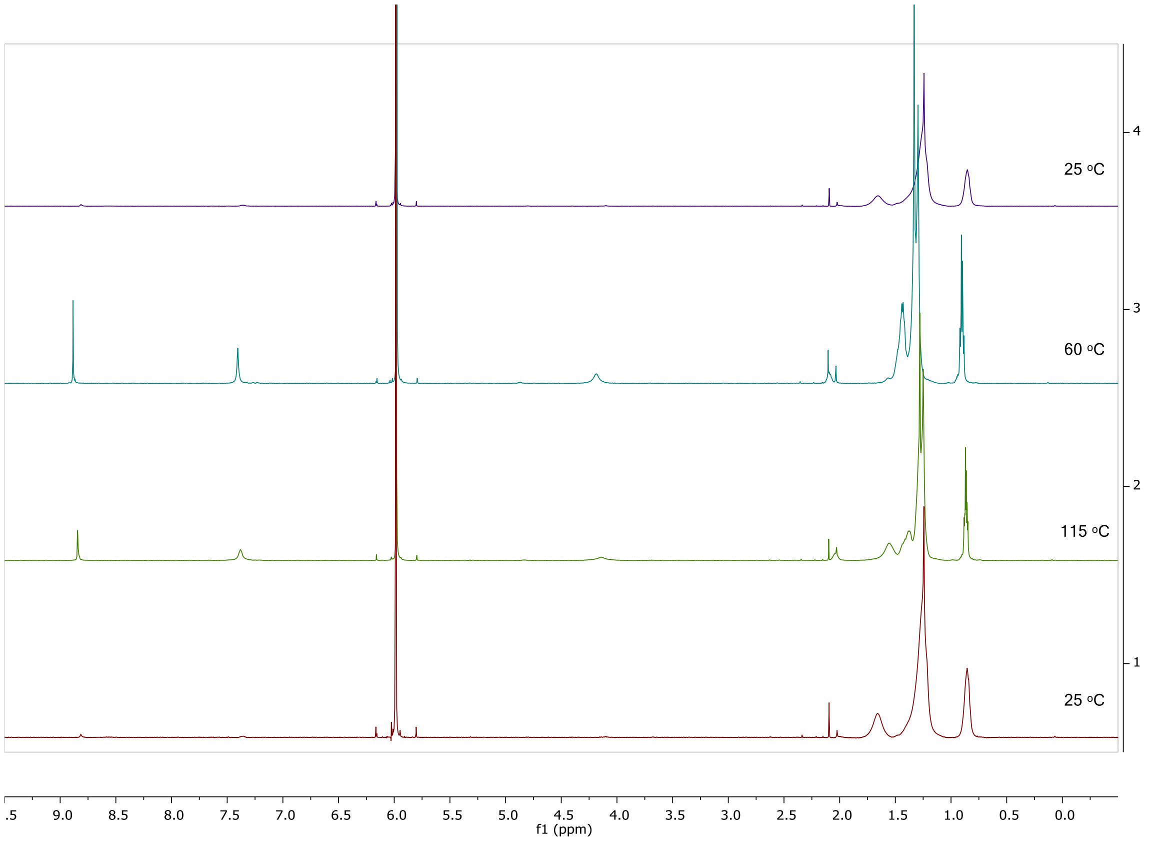

NMR spectrum of PNDIT2 in 1,1,2,2-Tetrachloroethane-d2 at 25 °C (purple; 4), 60 °C (blue; 3), 115 °C (green; 2), and back at 25 °C (red; 1).

DMP-PNDIT2

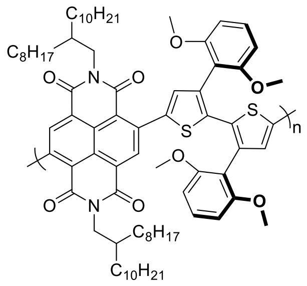

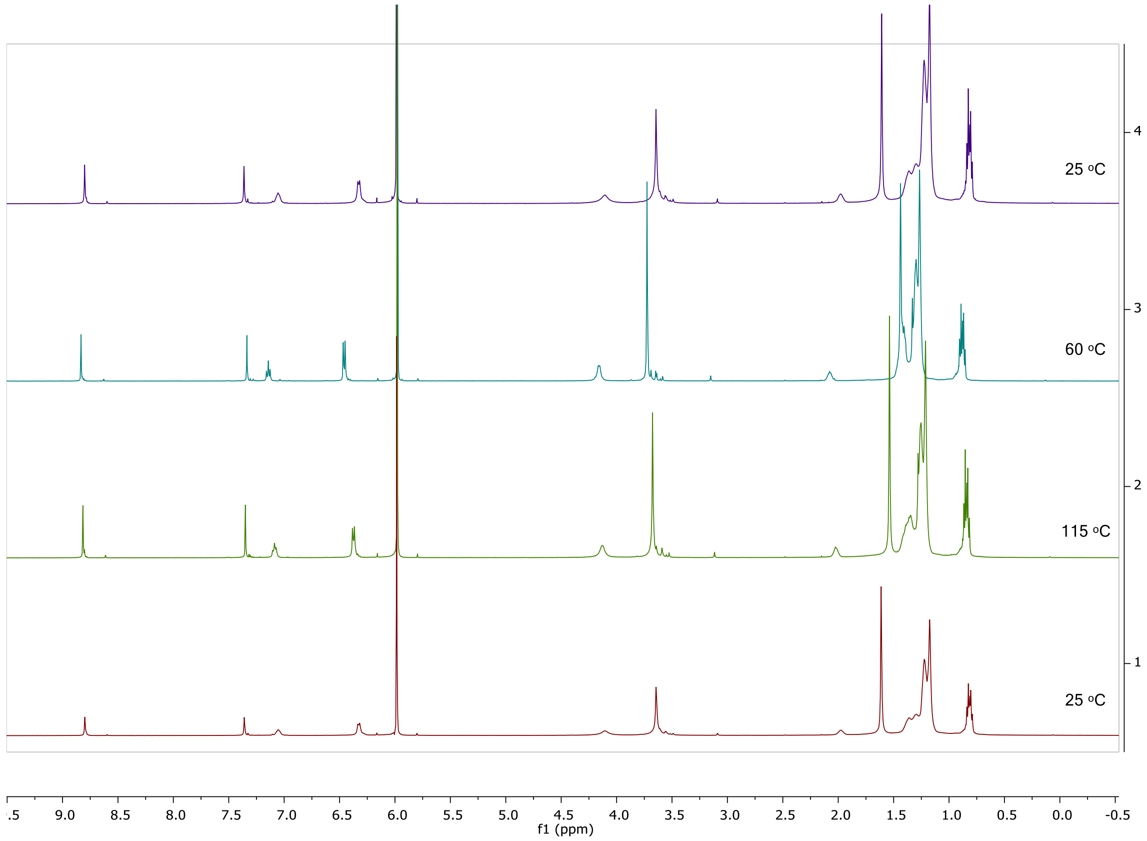

NMR spectrum of DMP-PNDIT2 in 1,1,2,2-Tetrachloroethane-d2 at 25 °C (purple; 4), 60 °C (blue; 3), 115 °C (green; 2), and back at 25 °C (red; 1).

E-PNDIT2

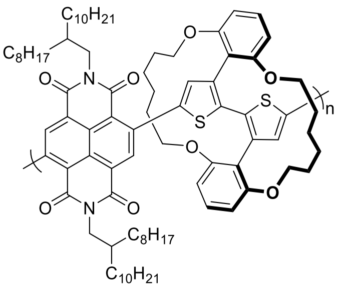

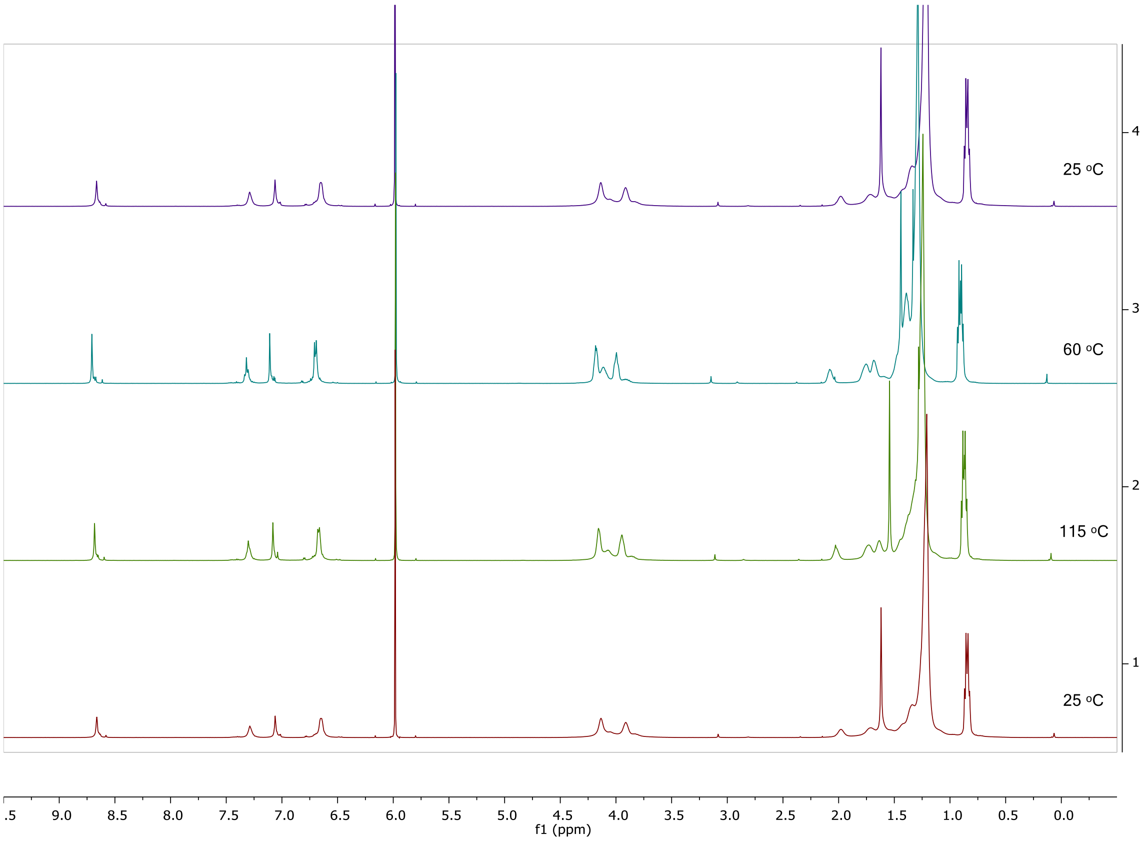

NMR spectrum of E-PNDIT2 in 1,1,2,2-Tetrachloroethane-d2 at 25 ºC (purple; 4), 60 ºC (blue; 3), 115 ºC (green; 2), and back at 25 ºC (red; 1).

2) DFT Calculations

- **<u>Dihedral angles</u> -** Calculated using b3lyp/6-31g*

PNDIT2

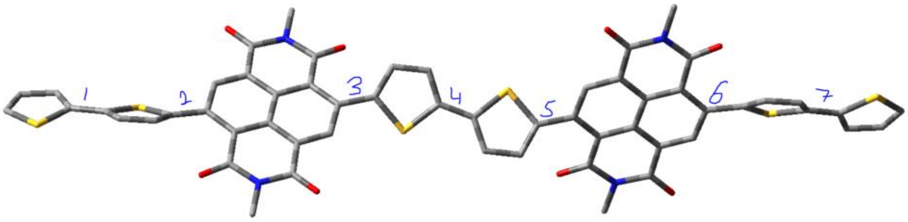

Position #	1	2	3	4	5	6	7
Angle °	161.5 (18.5)	142.9 (37.1)	134.7 (45.3)	163.4 (16.6)	133.2 (46.8)	135.5 (44.5)	161.5 (18.5)

Average dihedral angle between thiophenes (#1,4,7) = 162.13 (or 18.87).
Average dihedral angle between thiophene and NDI (#2,3,5,6) = 136.575 (or 43.425).

DMP-PNDIT2

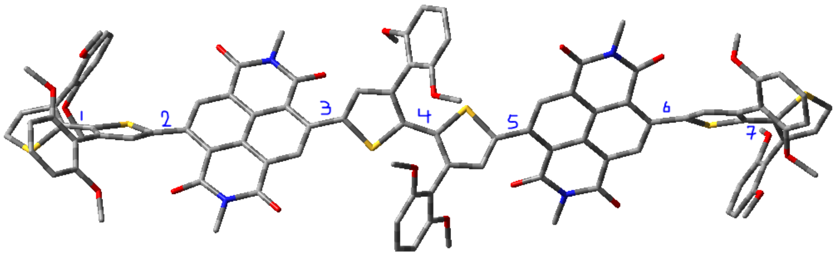

Position #	1	2	3	4	5	6	7
Angle °	150.95 (29.05)	130.53 (49.47)	126.78 (53.22)	152.46 (27.54)	131.02 (49.98)	133.60 (46.40)	150.37 (29.63)

Average dihedral angle between thiophenes (#1,4,7) = 151.26 (or 28.74).
Average dihedral angle between thiophene and NDI (#2,3,5,6) = 130.48 (or 49.52).

E-PNDIT2

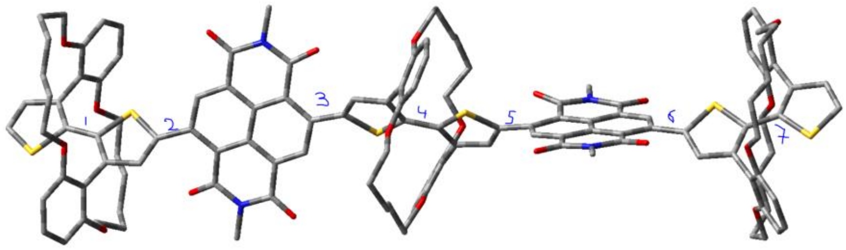

Position #	1	2	3	4	5	6	7
Angle °	177.7 (2.3)	128.8 (51.2)	135.3 (44.7)	177.5 (2.5)	130.3 (49.7)	133.4 (46.6)	176.4 (3.6)

Average dihedral angle between thiophenes (#1,4,7) = 177.2 (or 2.8).
Average dihedral angle between thiophene and NDI (#2,3,5,6) = 131.95 (or 48.05).

- **<u>HOMO/LUMO Levels</u> -** Calculated using b3lyp/6-31g*

PNDIT2

Eg = 1.98 eV

LUMO (-3.42 eV)

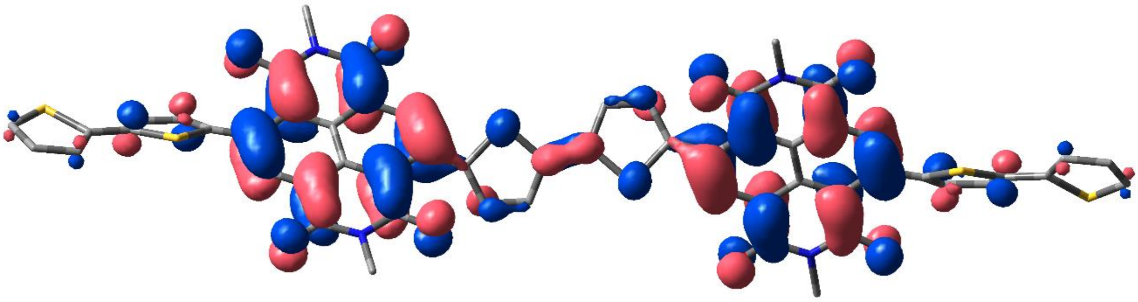

HOMO (-5.40 eV)

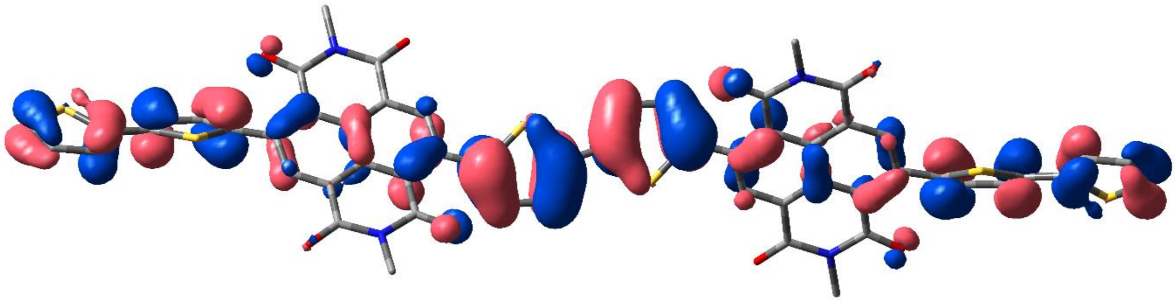

DMP-PNDIT2

Eg = 1.85 eV

LUMO (-3.12 eV)

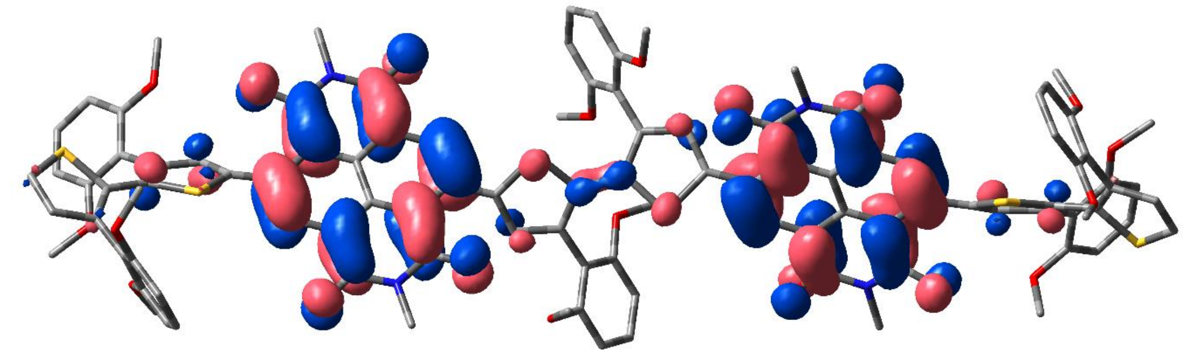

HOMO (-4.97 eV)

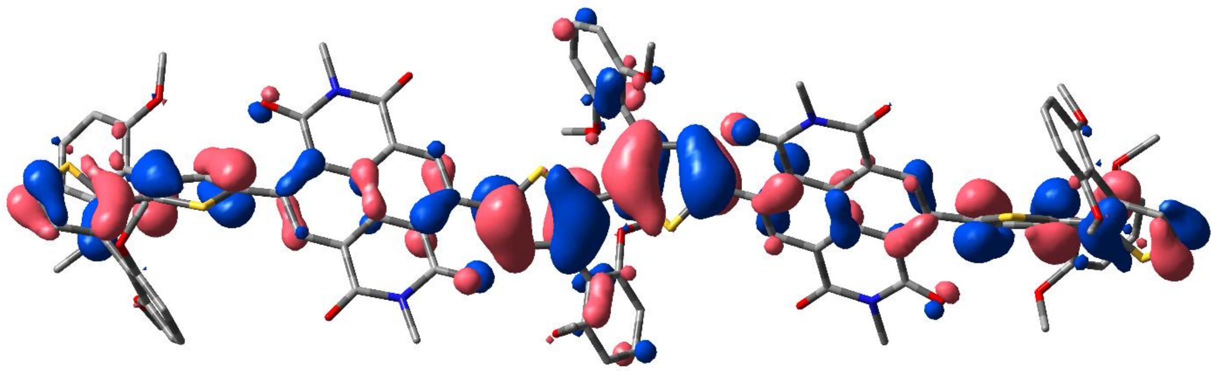

E-PNDIT2

Eg = 1.80 eV

LUMO (-3.12 eV)

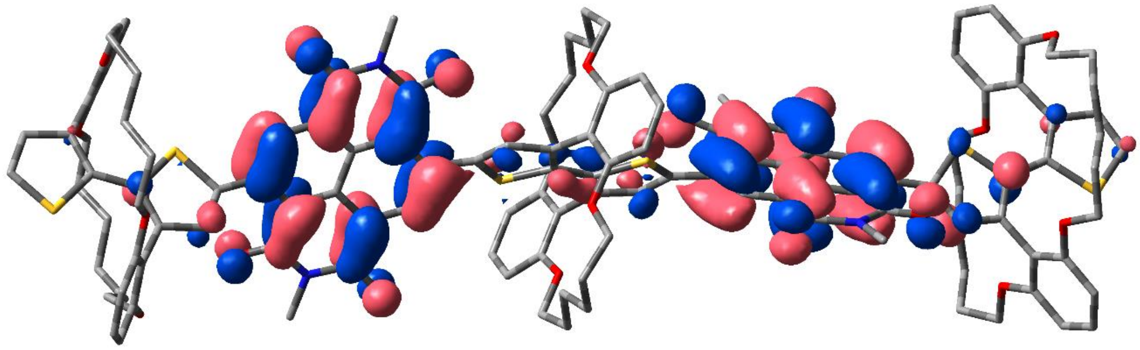

HOMO (-4.92 eV)

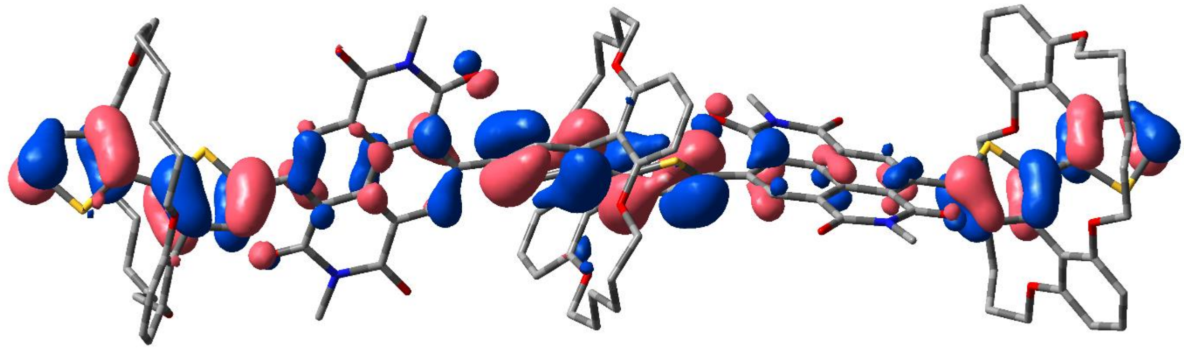

3) Thermal Characterization

Thermogravimetric Analysis (TGA) measurements (Figure S1) were performed with a TGA/DSC 1 STAR[e] System from Mettler Toledo, using alumina crucibles. All measurement were performed following the same TGA method: heating from 30°C to 600 °C with a 10°C/min rate. The measurements were performed under 50 mL/min N_2 flow.

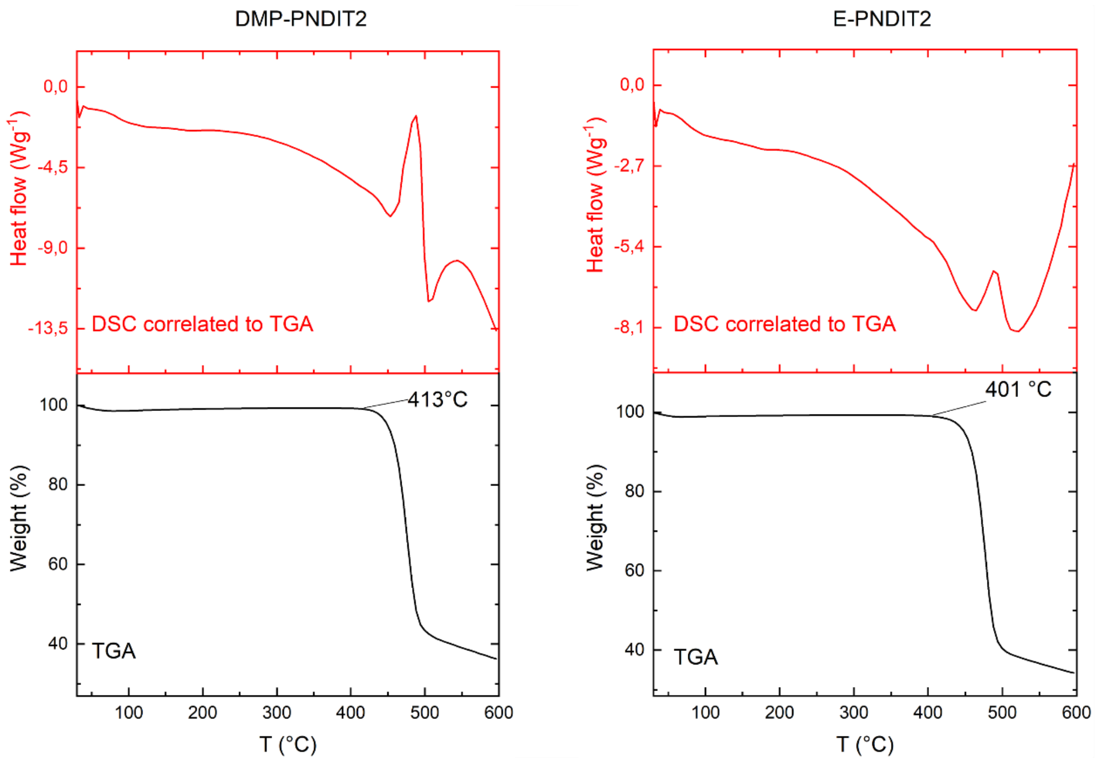

Figure S1. Thermogravimetric Analysis (TGA)

<u>DMP-PNDIT2</u>

Sample weight: 4.5047 mg

The thermogram shows significant weight loss and degradation after 413 °C. DSC analysis correlated to TGA shows no evident sign of decomposition before 400 °C as well.

Total weight loss at 600 °C: 64%

<u>E-PNDIT2</u>

Sample weight: 5.1840 mg

The thermogram shows significant weight loss and degradation after 401 °C. DSC analysis correlated to TGA shows no evident sign of decomposition before 400 °C as well.

Total weight loss at 600 °C: 66%

Differential Scanning Calorimetry (DSC) measurements (Figure S2) were performed on the polymers in powder form with a DSC 1 STAR^e System from Mettler Toledo, using aluminum crucibles. Calibration was performed with an Indium standard. All DSC measurements were performed following the same thermal treatment:

- 1st heating cycle: from 0 °C to 300 °C, 10°C/min
- isotherm at 300°C for 2 min.
- cooling: from 300 °C to 0°C, 10°C/min
- isotherm at 0°C for 2 min
- 2nd heating cycle: from 0 °C to 350°C, 10°C/min

The measurements were performed under 80 mL/min N_2 flow.

DMP-PNDIT2

Sample weight: 4.000 mg

Two endothermic peaks are present around 65 °C and 110 °C in the 1st heating cycle. Correspondent exothermic features are observed during the cooling cycle, suggesting melting and recrystallization processes during heating and cooling of the sample.

The transitions present above 240 °C in the heating cycles are probably related to instrumental artifacts. No sign of these features is present in DSC related to TGA, which support this hypothesis.

E-PNDIT2

Sample weight: 7.500 mg

A broad exothermic peak (result of peak integration: 37.77 mJ) is present between 140 °C and 250 °C in the first heating cycle. This feature is likely related to crystallization phenomena corresponding to the microstructural evolution seen in GIWAXS measurements (Figure S11 and Figure S12). No evident melting peak is present below 300 °C during the first heating cycle, which is compatible with GIWAXS data showing stable diffraction patterns till 300 °C. Similarly to DMP-PNDIT2, two endothermic features are present around 65 °C and 100 °C in the first heating cycle. These peaks are not present in the 2nd heating cycle, probably because a stable crystalline phase is formed with annealing above 140 °C, which is driven by inter-locking of the encapsulating rings (see discussion in the main text). Two noisy transitions around 255 °C and 325 °C are visible in the second heating cycle. These two features are probably instrumental artifacts. No sign of these features is present in DSC correlated to TGA (Figure S1), which support this hypothesis.

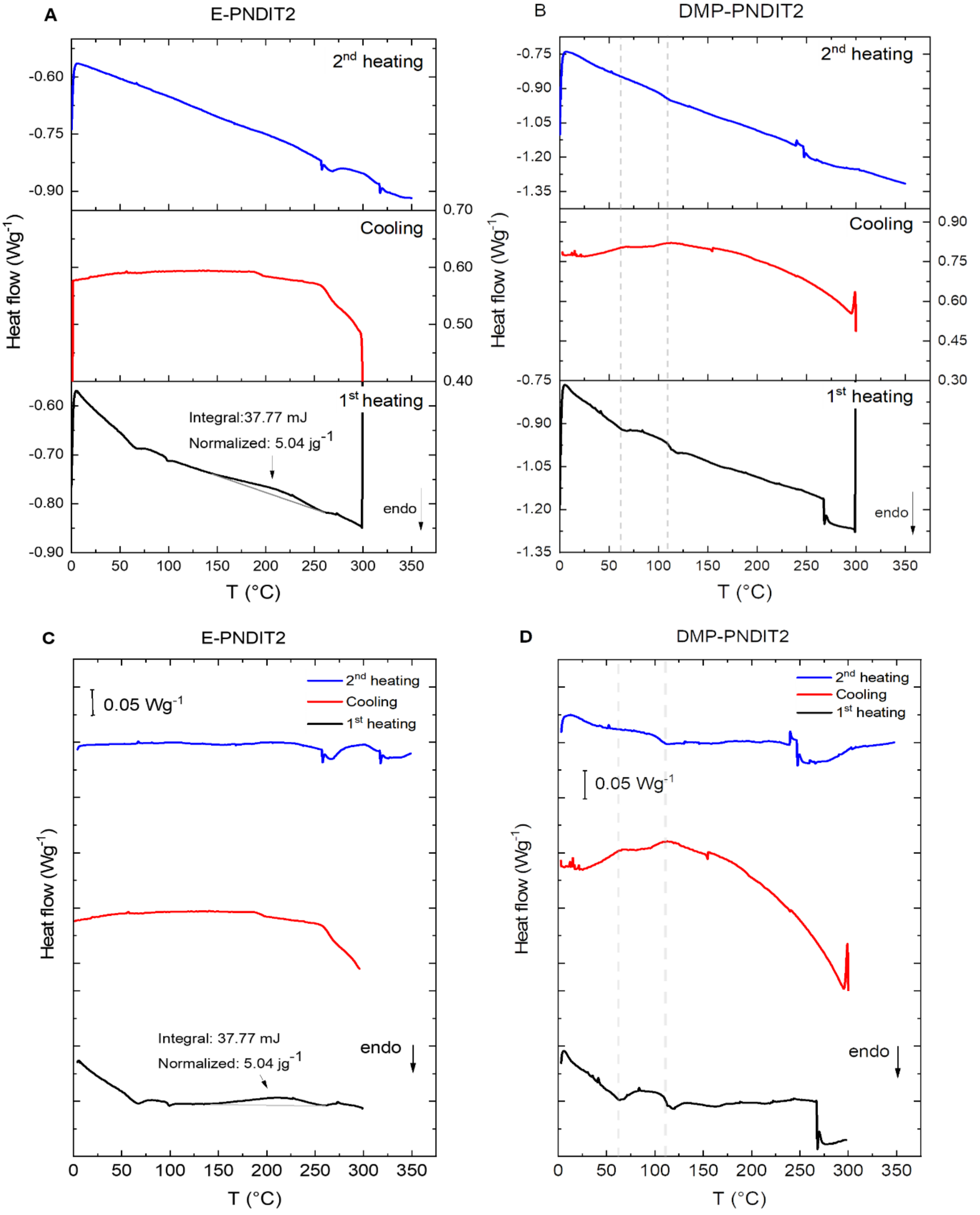

Figure S2. Differential Scanning Calorimetry (DSC). Top Graphs (A, B) show heating and cooling original cycles. Bottom graphs (C, D) show the same thermograms with horizontal baseline.

4) Optical Properties

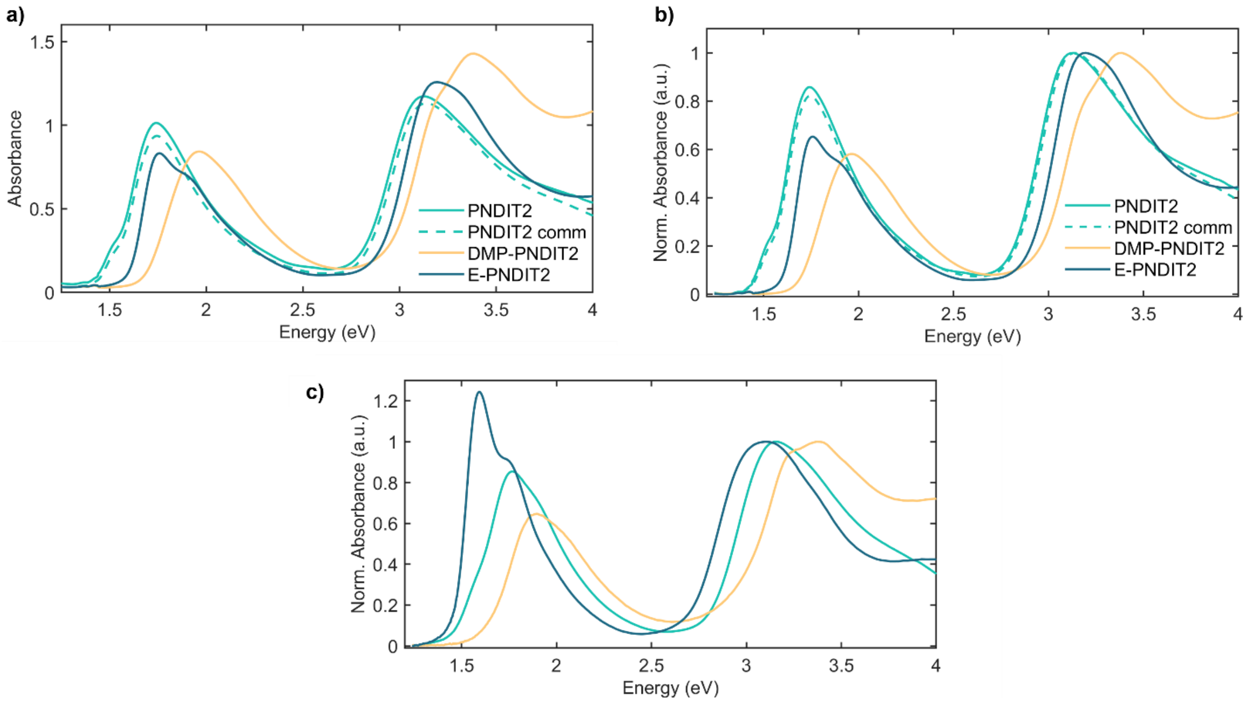

Figure S3. (a,b) UV-Vis absorption spectra of PNDIT2, DMP-PNDIT2 and E-PNDIT2 dissolved in toluene at a concentration of 0.1 g/L, and (c) in thin film. The spectra in (b) and (c) are normalized at the π-π* transition peaks.

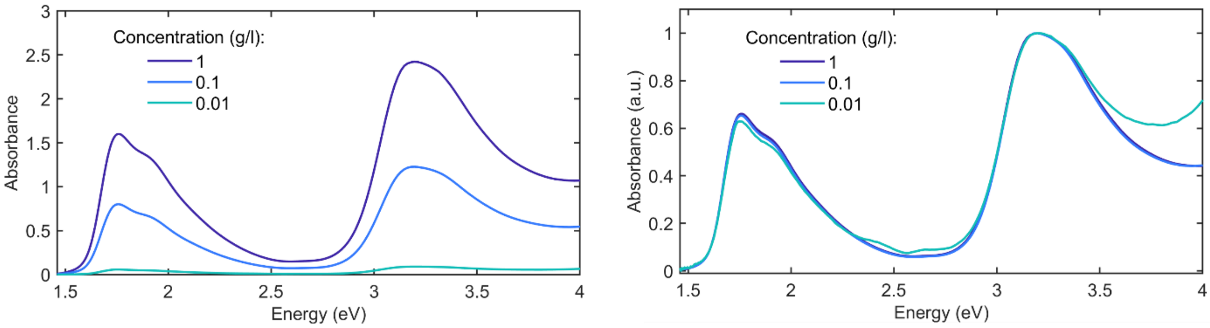

Figure S4. (a) Concentration dependent absorbance of E-PNDIT2 in toluene. (b) Normalization of the absorption spectra at the maximum of the high-energy absorption band (π-π* band).

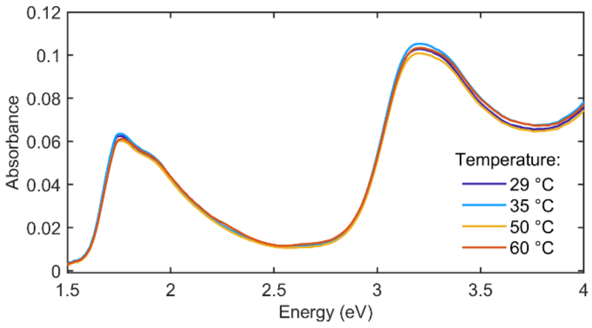

Figure S5. UV-Vis absorption of E-PNDIT2 (0.01 g/l in toluene) as function of the solution temperature.

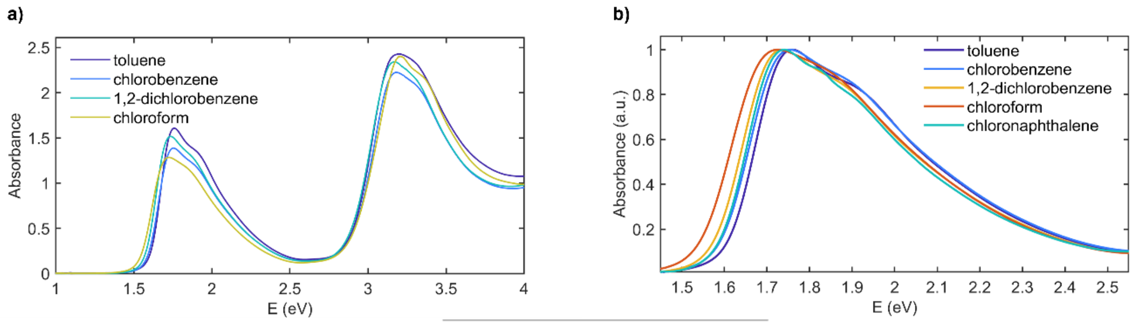

c)

**Solvent**	**E_T(30) (kcal mol^{-1})**
Toluene	33.9
Chlorobenzene	36.8
Chloronaphthalene	37.0
1,2-dichlorobenzene	38.0
Chloroform	39.1

Figure S6. (a) UV-Vis absorption spectra of E-PNDIT2 in solutions 1 g/l of various organic solvents. (b) Normalization of the absorption spectra at the maximum of the low energy absorption band (CT band). The redshift of the main peak and of the absorption tail are clear indication of solvatochromism. (c) Solvent polarity parameters E_T(30) for the employed solvents (from Ref. [4]).

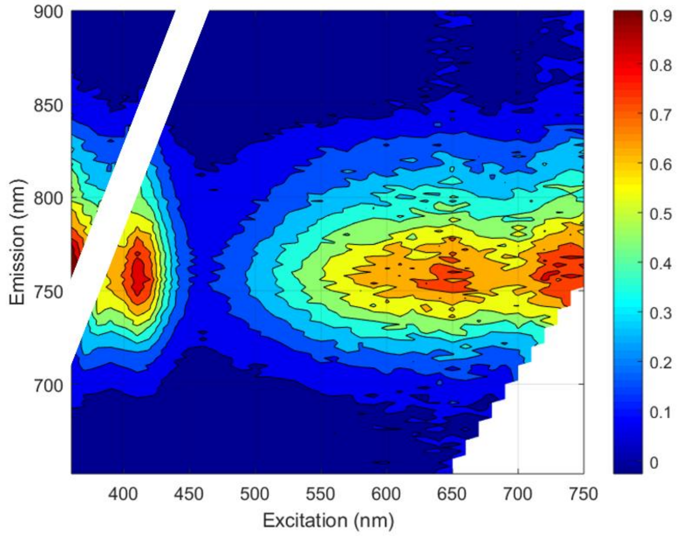

Figure S7. Excitation/emission map of E-PNDIT2 0.1 g/L in toluene.

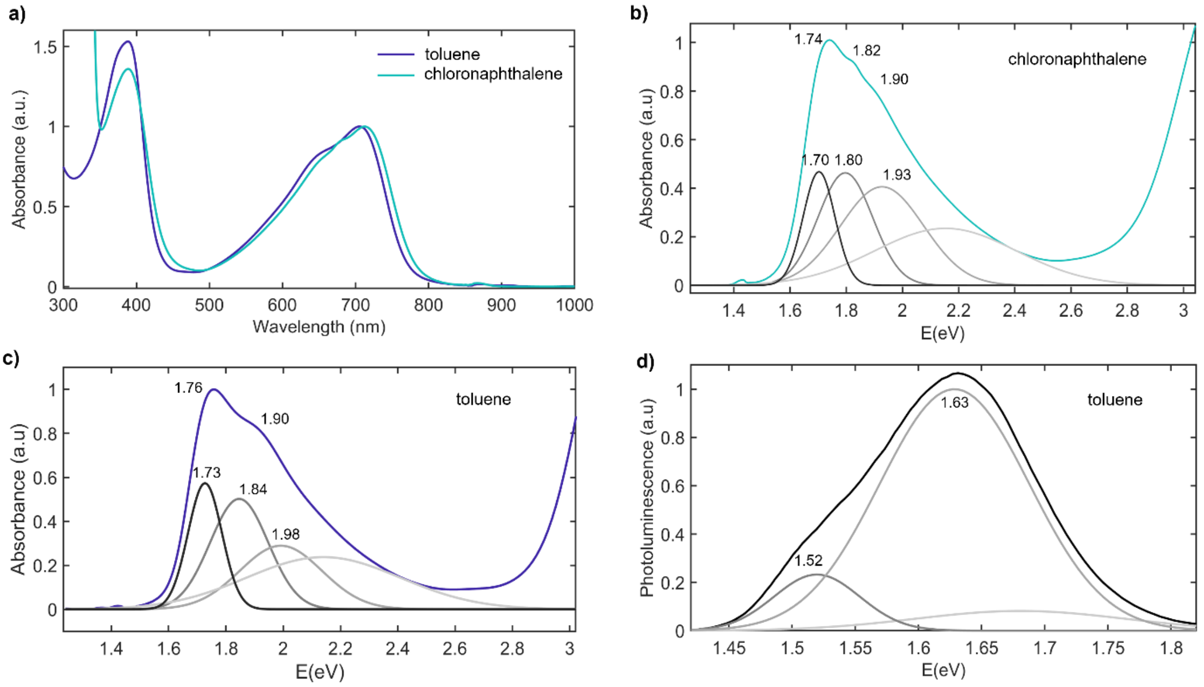

Figure S8. a) Comparison of the UV-Vis absorption spectra of E-PNDIT2 in toluene and in chloronaphthalene solutions (1 g/L), plotted as a function of the incident wavelength. **b,c)** Deconvolution with four gaussians of the absorption spectra in chloronaphthalene and toluene solutions, respectively. While the CT band of E-PNDIT2 in chloronaphthalene displays a fine structure with a main peak at 1.74 eV and two evident shoulders, a peak at 1.76 eV and only one shoulder are present in toluene solution. However, the deconvolution analysis shows that the best fit of the absorption

spectrum in both the solvents is described by three gaussians spaced of ~0.12 eV (compatible with vibronic coupling involving collective stretching modes found in virtually all π-conjugated systems[5,6]). **d)** Emission spectrum (excitation wavelength: 650 nm) of E-PNDIT2 in toluene (0.1 g/L), and its deconvolution with three gaussians. Two gaussians at 1.52 eV and 1.63 eV mainly contribute to the emission spectrum. The energy spacing between the peaks is of 0.11 eV, in good agreement with the spacing derived by deconvolution of the absorption spectra. Therefore, such a fine structure both in the absorption and emission spectra clearly indicates vibronic progression of unaggregated chains, as opposed to different aggregates.

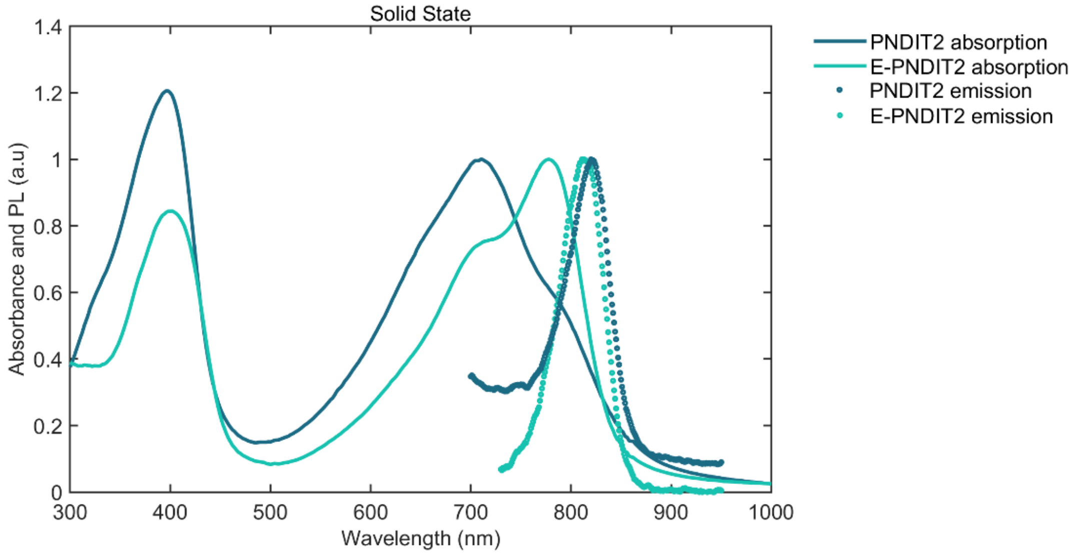

Figure S9. Comparison of the normalized absorption and photoluminescence (PL) spectra of thin films of PNDIT2 and E-PNDIT2.

5) Thin Film Microstructure

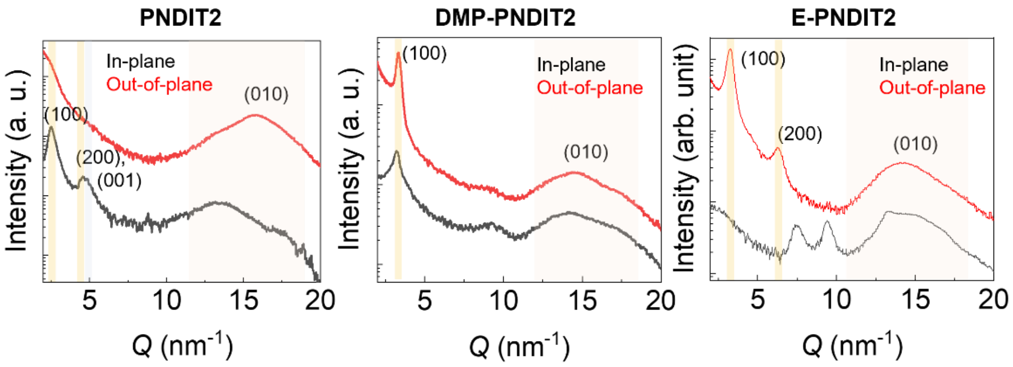

Figure S10. In-plane/out-of-plane integrated diffraction patterns of PNDIT2, DMP-PNDIT2 and E-PNDIT2.

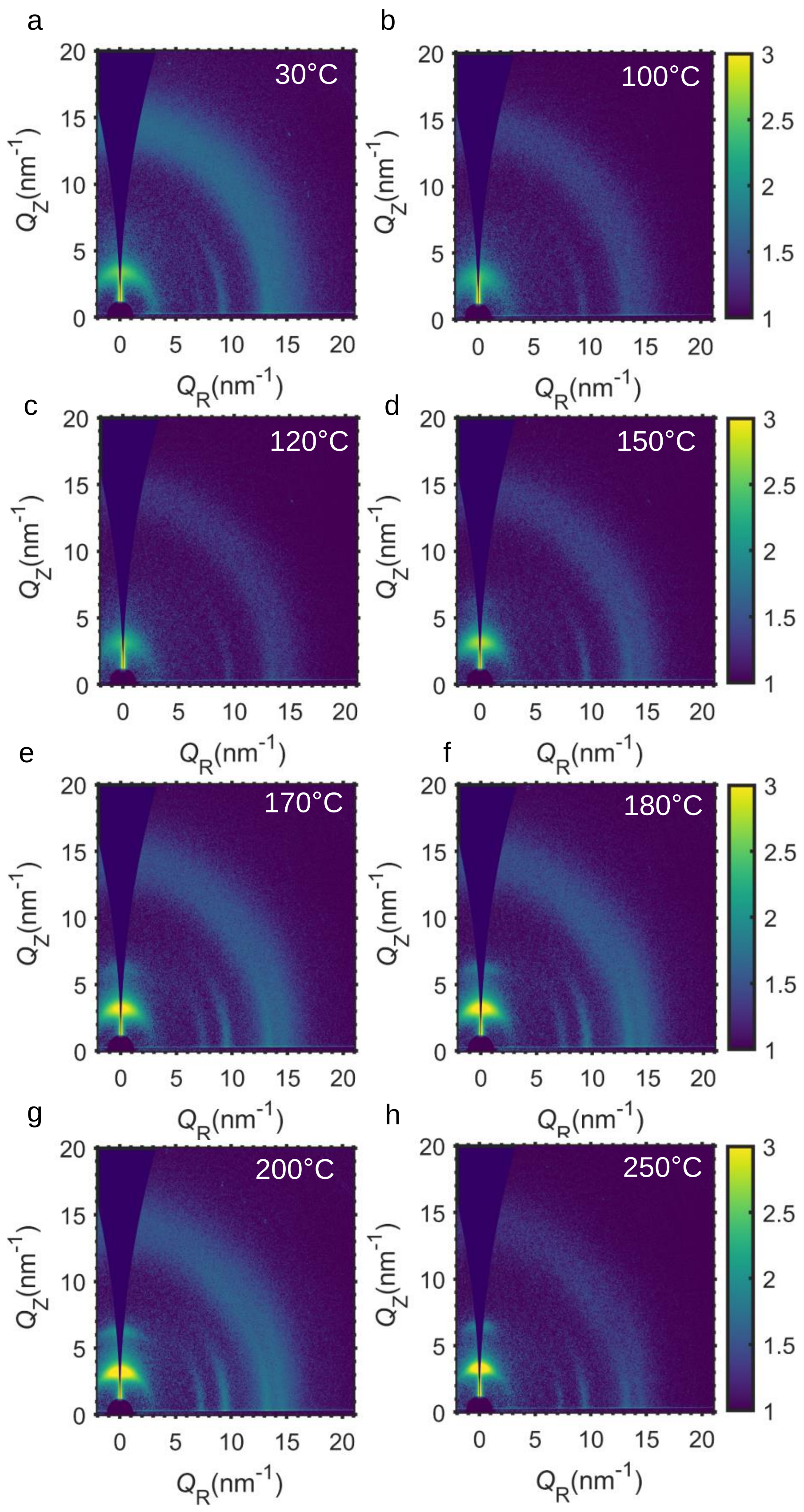

Figure S11. *In-situ* GIWAXS while annealing a film of E-PNDIT2, which has not been previously annealed.

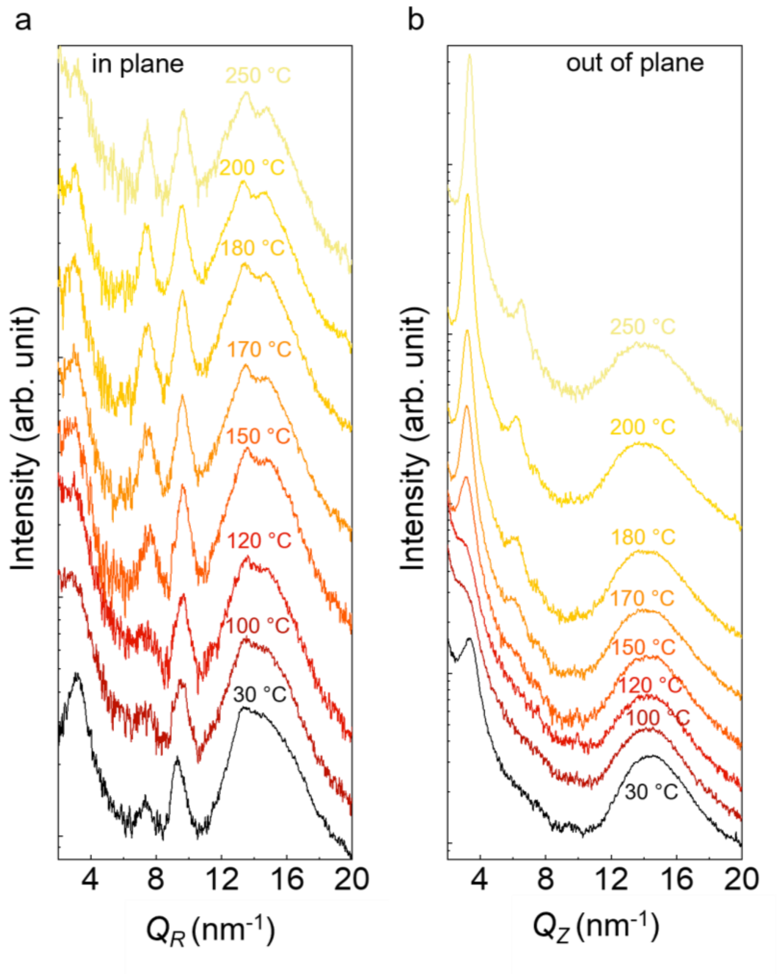

Figure S12. In-plane/out-of-plane integrated diffraction patterns of E-PNDIT2 (from 2D patterns of Figure S11).

6) Charge Transport

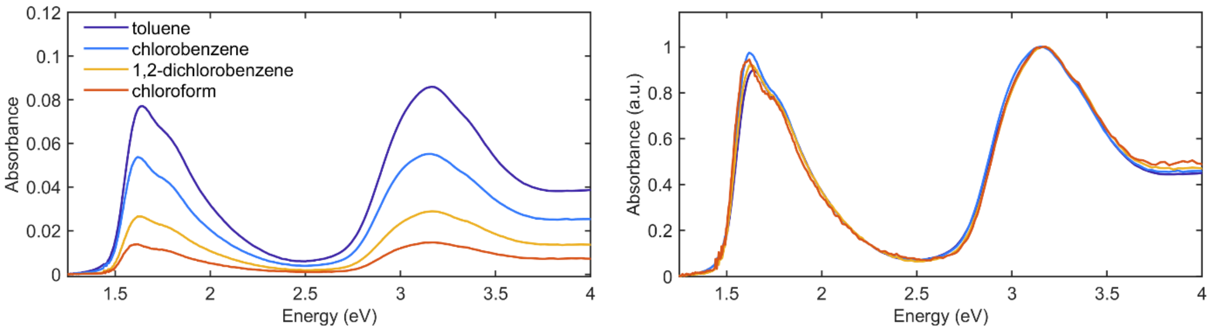

Figure S13. UV-Vis absorption of thin films of E-PNDIT2 deposited from various solvent solutions at a concentration of 5 g/L

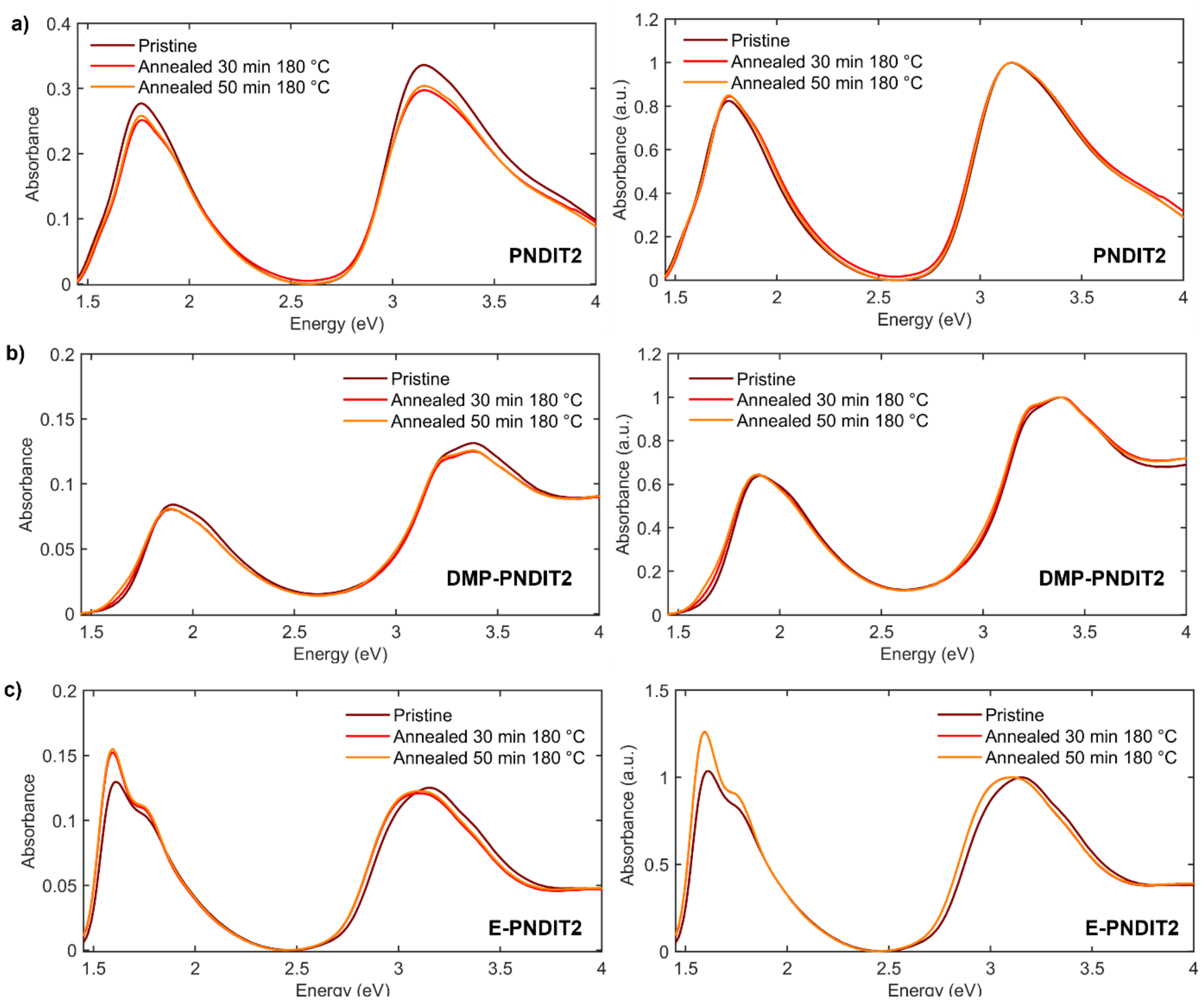

Figure S14. Effect of the annealing temperature on the UV-Vis absorption spectra of (a) PNDIT2, (b) DMP-PNDIT2 and (c) E-PNDIT2.

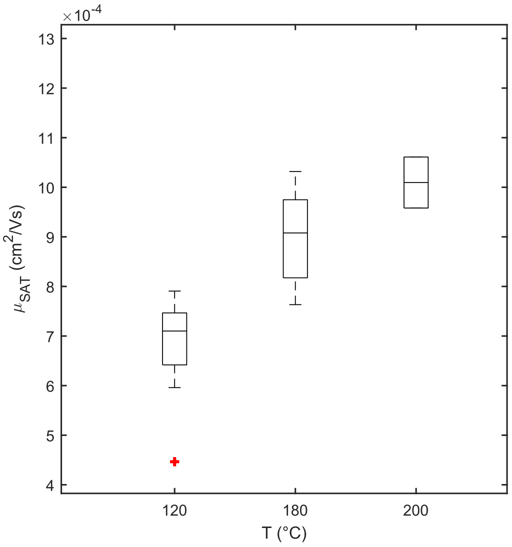

Figure S15. Box-plot of the saturation mobility of devices based on E-PNDIT2 films annealed for 30 min at different temperatures (spin-casted from toluene solution at a concentration of 10 g/L).

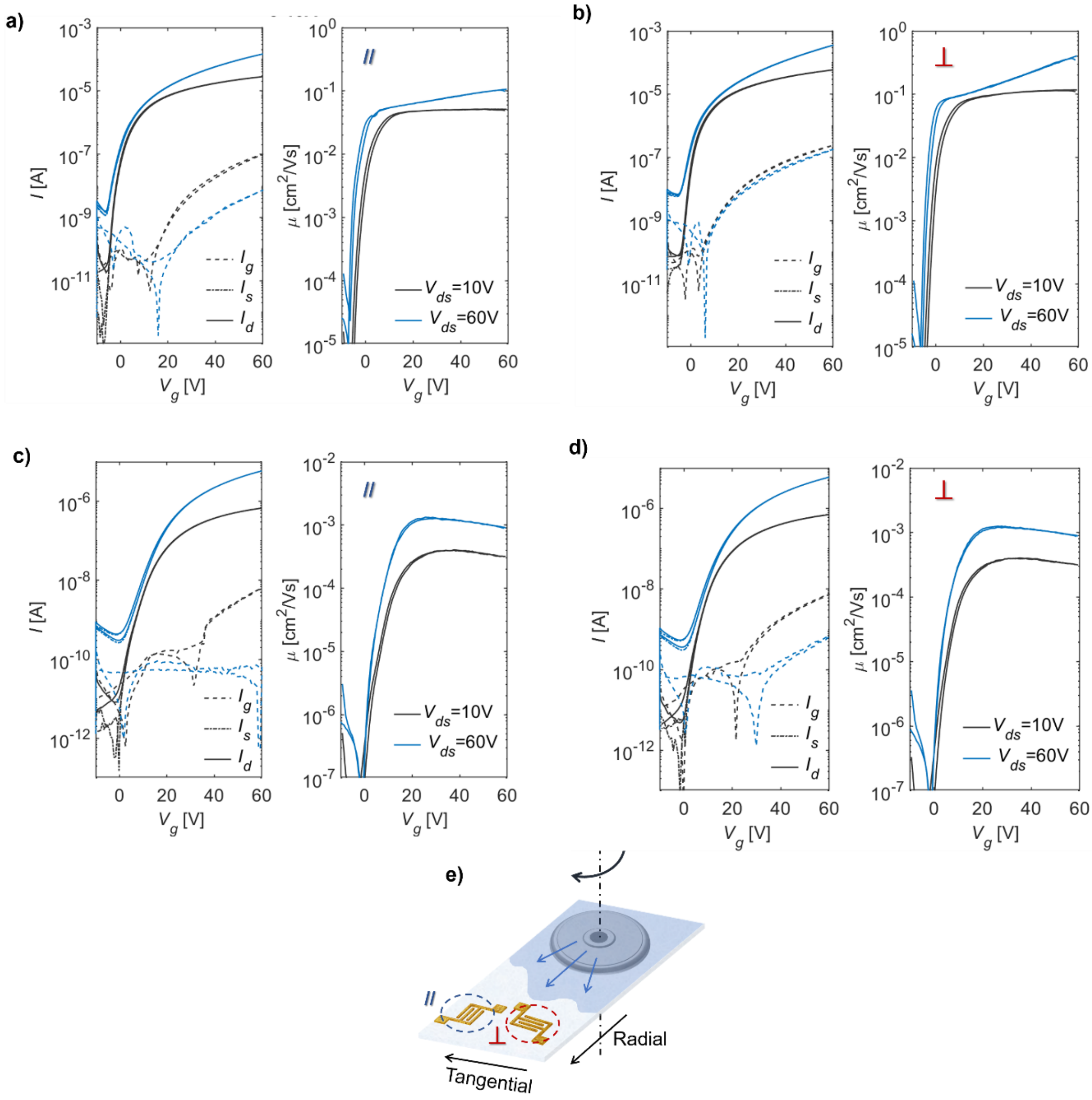

Figure S16. Transfer curves, and related mobility curves as a function of the gate potential, of devices based on PNDIT2 (a,b) and on E-PNDIT2 (c, d). Source and drain contacts are oriented parallel to the alignment direction in panels (a,c), and perpendicularly in panels (b,d). PNDIT2-based devices have L = 10 μm and W = 2 mm, E-PNDIT2-based devices L = 2.5 μm and W = 2 mm. e) Scheme of the off-center spin-coating.

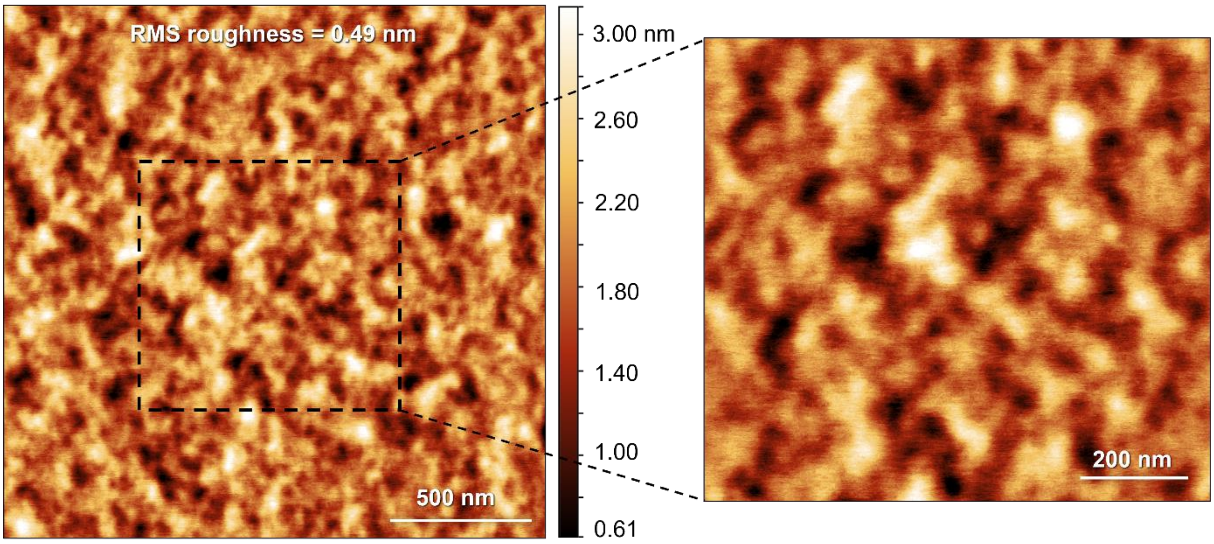

Figure S17. AFM topography of E-PNDIT2 thin-film.

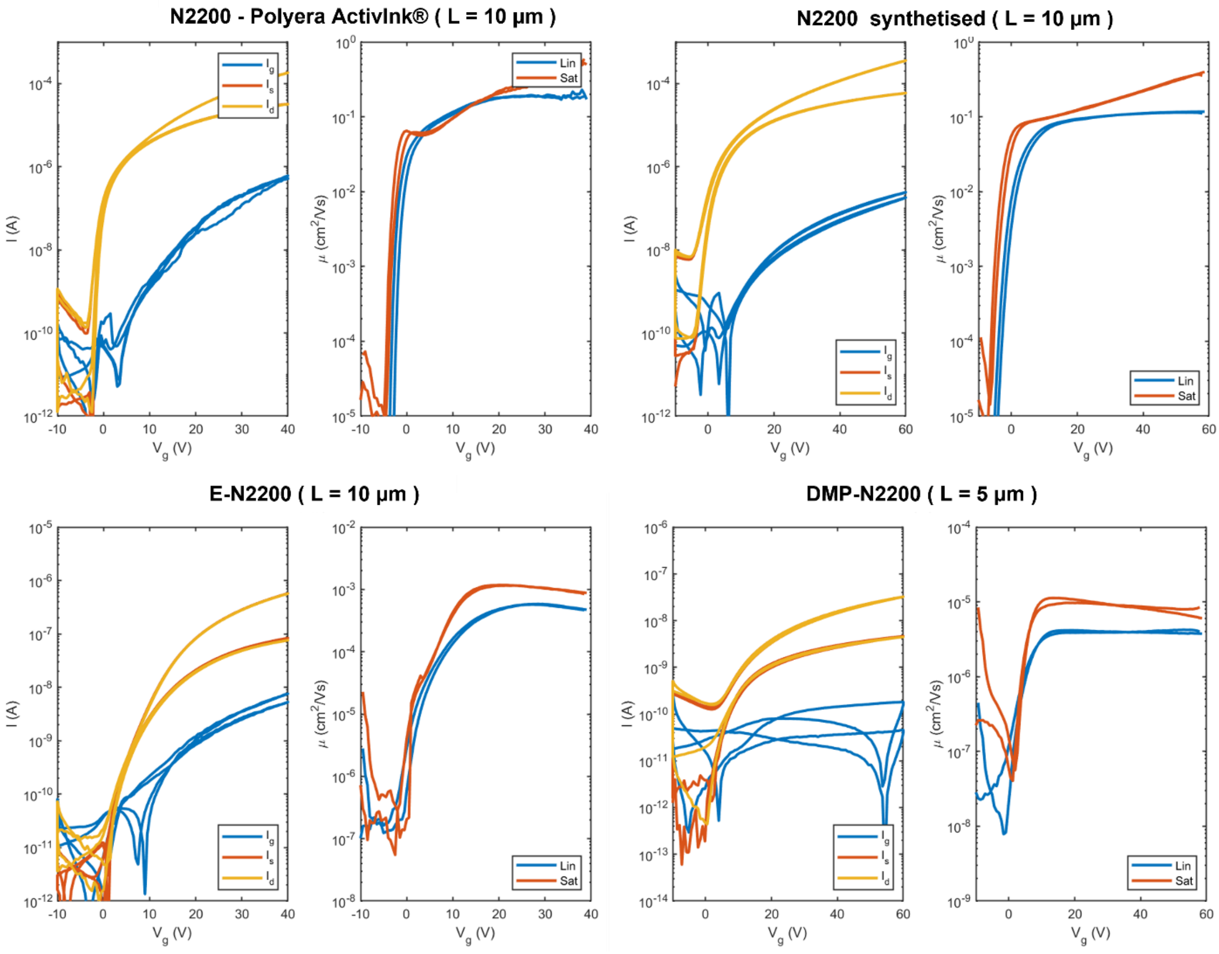

Figure S18. Transfer curves, and related mobility curves as a function of the gate potential, of devices based on PNDIT2 (both commercial ink and synthesized in this work), E-PNDIT2 and DMP-PNDIT2.

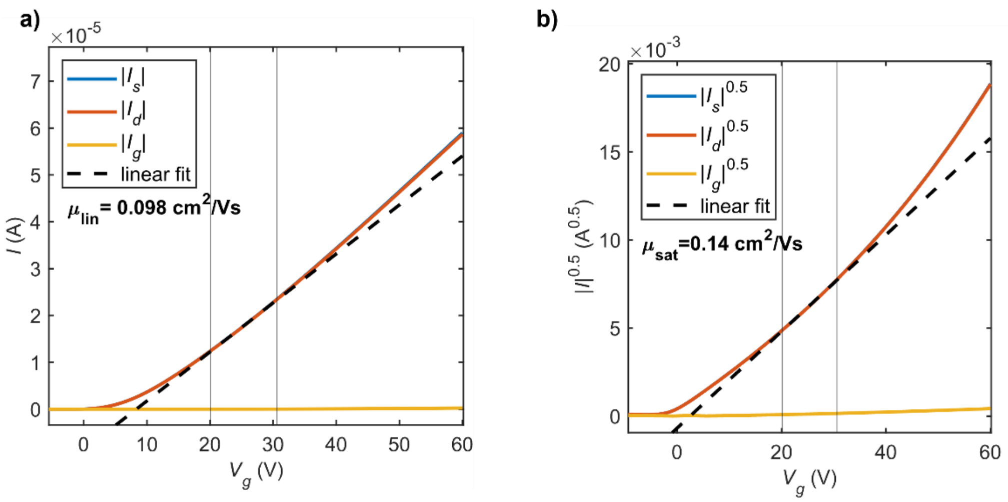

Figure S19. Mobility extraction from the linear slope of the transistors transfer curves a) in linear regime (V_{ds} = 10 V), and b) in saturation regime (V_{ds} = 60 V). The slope is obtained by fitting the curves in the range V_g = 20 - 30 V.

References

(1) Royakkers, J.; Guo, K.; Toolan, D. T. W.; Feng, L. W.; Minotto, A.; Congrave, D. G.; Danowska, M.; Zeng, W.; Bond, A. D.; Al-Hashimi, M.; Marks, T. J.; Facchetti, A.; Cacialli, F.; Bronstein, H. Molecular Encapsulation of Naphthalene Diimide (NDI) Based π-Conjugated Polymers: A Tool for Understanding Photoluminescence. *Angew. Chemie - Int. Ed.* **2021**, *60* (47), 25005–25012. https://doi.org/10.1002/anie.202110139.

(2) Sugiyasu, K.; Honsho, Y.; Harrison, R. M.; Sato, A.; Yasuda, T.; Seki, S.; Takeuchi, M. A Self-Threading Polythiophene: Defect-Free Insulated Molecular Wires Endowed with Long Effective Conjugation Length. *J. Am. Chem. Soc.* **2010**, *132* (42), 14754–14756. https://doi.org/10.1021/ja107444m.

(3) Chen, Z.; Zheng, Y.; Yan, H.; Facchetti, A. Naphthalenedicarboximide- vs Perylenedicarboximide-Based Copolymers. Synthesis and Semiconducting Properties in Bottom-Gate N-Channel Organic Transistors. *J. Am. Chem. Soc.* **2009**, *131* (1), 8–9. https://doi.org/10.1021/ja805407g.

(4) Reichardt, C. Solvatochromic Dyes as Solvent Polarity Indicators. *Chem. Rev.* **1994**, *94* (8), 2319–2358. https://doi.org/10.1021/cr00032a005.

(5) Qarai, M. B.; Chang, X.; Spano, F. C. Vibronic Exciton Model for Low Bandgap Donor-Acceptor Polymers. *J. Chem. Phys* **2020**, *153*, 244901. https://doi.org/10.1063/5.0029193.

(6) Denti, I.; Cimò, S.; Brambilla, L.; Milani, A.; Bertarelli, C.; Tommasini, M.; Castiglioni, C. Polaron Confinement in N-Doped P(NDI2OD-T2) Unveiled by Vibrational Spectroscopy. *Chem. Mater.* **2019**, *31* (17), 6726–6739. https://doi.org/10.1021/acs.chemmater.9b01218.
